# A pH responsive nanocomposite for combination sonodynamic‐immunotherapy with ferroptosis and calcium ion overload via SLC7A11/ACSL4/LPCAT3 pathway

**DOI:** 10.1002/EXP.20240002

**Published:** 2024-06-26

**Authors:** Xue Bai, Jun Kang, Silong Wei, Yun Wang, Yangsui Liu, Bo Yuan, Qian Lu, Huansong Li, Jun Yan, Xi Yang, Jin Chang

**Affiliations:** ^1^ School of Life Sciences Tianjin University Tianjin China; ^2^ Tianjin Key Laboratory of Function and Application of Biological Macromolecular Structures School of Life Sciences Tianjin University Tianjin China; ^3^ Department of Biological Sciences University of Toronto Scarborough Toronto Canada; ^4^ Chen Guanxing Dental Clinic Ningbo China; ^5^ Hepatobiliary Pancreatic Center Xuzhou Central Hospital Xuzhou China; ^6^ Hepatopancreatobiliary Center Beijing Tsinghua Changgung Hospital Tsinghua University Beijing China; ^7^ School of Clinical Medicine Tsinghua University Beijing China; ^8^ Department of Oral Maxillofacial‐Head and Neck Oncology Shanghai Ninth People's Hospital, Shanghai Jiao Tong University School of Medicine, College of Stomatology, Shanghai Jiao Tong University Shanghai China

**Keywords:** ferroptosis, parthenolide, sonodynamic therapy, tumor immunotherapy

## Abstract

Sonodynamic therapy offers a non‐invasive approach to induce the death of tumor cells. By harnessing ultrasound waves in tandem with sonosensitizers, this method produces reactive oxygen species (ROS) that inflict oxidative damage upon tumor cells, subsequently causing their demise. Ferroptosis is a regulatory form of cell death that differs from other forms, characterized by iron accumulation, ROS accumulation, and lipid peroxidation. In the presented research, a nanoparticle formulation, parthenolide/ICG‐CaCO_3_@lipid (PTL/ICG‐CaCO_3_@Lip), has been engineered to amplify ferroptosis in tumor cells, positioning it as a potent agent for sonodynamic cancer immunotherapy. This nanoparticle significantly augments ROS levels within tumor cells, inducing oxidative stress that leads to cell death. The therapeutic potential of PTL/ICG‐CaCO_3_@Lip, both in vivo and in vitro, has been convincingly demonstrated. Furthermore, RNA‐seq analysis insights revealed that PTL/ICG‐CaCO_3_@Lip facilitates tumor cell ferroptosis by regulating P53 to downregulate SLC7A11 protein expression, thereby inhibiting the glutamate‐cystine antiporter system Xc^−^ and stimulating ACSL4/LPCAT3 pathways. This pioneering work uncovers an innovative strategy for combatting tumors, leveraging enhanced oxidative stress to promote cell ferroptosis, and paves the way for groundbreaking cancer immunotherapeutic interventions.

## Graphical abstract

In this research, a nanoparticle formulation, Parthenolide/ICG‐CaCO3@Lipid (PTL/ICG‐CaCO3@Lip), has been engineered to amplify ferroptosis in tumor cells, positioning it as a potent agent for sonodynamic cancer immunotherapy. This nanoparticle significantly augments ROS levels within tumor cells, inducing oxidative stress, and regulating P53 to downregulate SLC7A11 protein expression, thereby inhibiting the glutamate‐cystine antiporter system Xc‐ and stimulating ACSL4/LPCAT3 pathways.

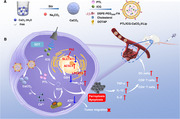

## 1 INTRODUCTION

Cancer, a multifaceted disease, poses a serious threat to human life and health^[^ ^1^ ^]^. Currently, the advent of cancer immunotherapy has provided new means of combating tumors. By stimulating and amplifying the body's own immune response,^[^ ^2^ ^]^ immunotherapy—encompassing immune checkpoint inhibitors, cancer vaccines, and adoptive cell therapy—has demonstrated encouraging outcomes in the battle against cancer,^[^ ^3^ ^]^ establishing itself as the main means to conquer tumors.

Sonodynamic therapy (SDT) is a non‐invasive treatment modality that utilizes ultrasound and a photosensitizer to induce cell death in cancer cells^[^ ^4^, ^5^ ^]^. Thanks to its high penetration, precise targeting, and robust safety profile, SDT has gained significant traction in the realm of cancer treatment^[^ ^6^, ^7^ ^]^. This process works by generating reactive oxygen species (ROS), which induce oxidative damage in tumor cells, ultimately causing their demise^[^ ^8^ ^]^. ROS are byproducts of cellular aerobic metabolism, including hydroxyl radicals (•OH), superoxide anion (O_2_•–), singlet oxygen (^1^O_2_), and hydrogen peroxide (H_2_O_2_) ^[^ ^9^, ^10^ ^]^. They play pivotal roles in various biological processes, such as the cell cycle, apoptosis, autophagy, and immunity^[^ ^11^, ^12^ ^]^. However, when produced in excess, ROS can inflict oxidative damage on cellular DNA, proteins, and lipids, further leading to cell death^[^ ^13^, ^14^ ^]^.

Ferroptosis is a ferrous ion dependent, ROS mediated regulatory form of cell death^[^ ^15^, ^16^ ^]^, distinct from other forms of cell death, characterized by iron accumulation, ROS accumulation, and lipid peroxidation^[^ ^17^, ^18^ ^]^. For Ferroptosis, ferrous ions will catalyze high levels of endogenous hydrogen peroxide (H_2_O_2_) to produce ROS through the Fenton reaction, leading to lipid peroxidation and inducing cancer cell death^[^ ^19^, ^20^, ^21^, ^22^ ^]^. This unique cell death mechanism has attracted widespread attention in the field of cancer research due to its potential as a therapeutic target. Parthenolide (PTL) is a natural sesquiterpene lactone isolated from the Asteraceae family^[^ ^23^ ^]^. In recent years, PTL has been extensively used in the field of tumor treatment, such as melanoma, lung cancer, breast cancer, gastric cancer, and bile duct cancer^[^ ^24^, ^25^, ^26^, ^27^, ^28^ ^]^. The key anti‐tumor mechanism of PTL is to induce the production of ROS and cause damage to mitochondrial membrane potential^[^ ^29^, ^30^ ^]^. However, due to the drawbacks of weak water solubility, relative instability under chemical and physiological conditions, and low oral bioavailability, PTL's clinical application is still hindered^[^ ^31^, ^32^, ^33^ ^]^.

**SCHEME 1:**
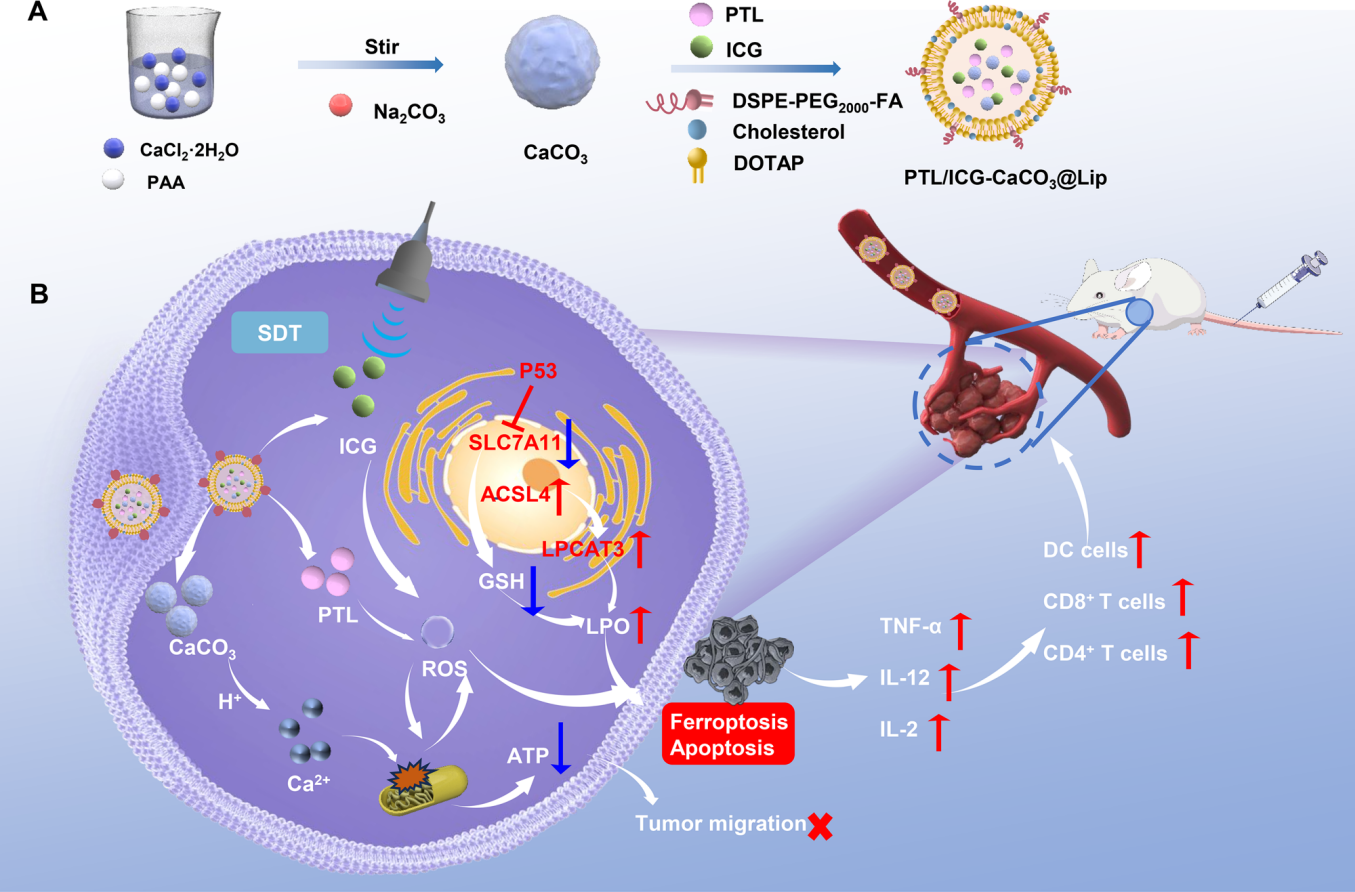
Schematic diagram of PICL preparation and tumor immunotherapy application. (A) The synthesis process of PICL. (B) The mechanism of PICL used in tumor immunotherapy.

Therefore, this study proposes a liposome‐based nanomedicine (PTL/ICG‐CaCO_3_@Lip, PICL) designed for cancer immunotherapy. In the acidic tumor microenvironment, PICL releases PTL and the sonosensitizer indocyanine green (ICG), disrupting the physiological metabolism of the tumor cells and generating a significant amount of ROS, leading to oxidative damage. Concurrently, Ca^2+^ disrupt the mitochondrial balance, inflicting mitochondrial damage. This synergistic action induces ferroptosis and apoptosis in the tumor cells (Scheme 1). The remarkable anti‐cancer efficacy of PICL is thoroughly demonstrated in both in vitro and in vivo experiments. Notably, transcriptomic analysis further substantiates that PICL can induce ferroptosis in cells, thereby exerting its anti‐cancer effects. Hence, this research presents a novel strategy for cancer treatment that involves amplifying oxidative stress to promote ferroptosis in cells.

## 2 MATERIALS AND METHODS

### 2.1 Materials

The sodium carbonate (Na_2_CO_3_) was purchased from Heowns (Tianjin, China). The poly (acrylic acid) (PAA, Mw 2000) and cholesterol were purchased from Aladdin. The calcium chloride dihydrate (CaCl_2_•2H_2_O) was obtained from Macklin (Shanghai, China). 1,2‐dioleoyl‐3‐trimethylammonium‐propane chloride (DOTAP) was obtained from MedChemExpress (Shanghai, China). DSPE‐PEG_2000_‐FA was purchased from shyuanye (Shanghai, China), and PTL was purchased from push‐herbchem (Chengdu, China). ICG was bought from Meryer (Shanghai, China). 1,3‐diphenylisobenzofuran (DPBF) was obtained from jkchemical (Beijing, China). RPMI 1640 medium, phosphate‐buffered solution (PBS), fetal bovine serum (FBS), and 0.25% trypsin‐EDTA were purchased from Gibco. ACSL4/FACL4 Polyclonal antibody, LPCAT3 Monoclonal antibody, GAPDH Monoclonal antibody were purchased from Proteintech.

### 2.2 Cells and animals

The 4T1 cells, B16F10 cells, and HEK 293T cells were cultivated in RPMI 1640 medium, DMEM f12 medium, and DMEM high glucose medium rich in FBS (10%, v/v), respectively, under humidified conditions at 37°C with 5% CO_2_. Female BALB/c mice were obtained from the Beijing Vital River Laboratory Animal Technology Co., Ltd. (Beijing, P. R. China) and reared under aseptic conditions. Animal welfare and experimental procedures were approved by the Institutional Animal Care and Use Committee of Tianjin University (Approval No. TJUE‐2023‐242).

### 2.3 Synthesis of CaCO_3_ nanoparticles

CaCO_3_ nanoparticles were synthesized using chemical co‐precipitation method. Initially, 114.0 mg of PAA was incorporated into a CaCl_2_ solution (10 mL, 0.2 m), mix and stir at 700–800 rpm for 1 h. Afterward, a Na_2_CO_3_ solution (10 mL, 0.2 m) was added, and the mixture was stirred for an additional hour. The product was then dialyzed for 24 h using a dialysis bag to thoroughly remove PAA and any unreacted substances.

### 2.4 Synthesis of PTL/ICG‐CaCO_3_@Lip NPs

PTL/ICG‐CaCO_3_@Lip NPs (PICL) was prepared using the thin‐film hydration method. PTL (20 µL, 200 mm), DOTAP (200 µL, 25 m), cholesterol (15 µL, 0.1 m), and DSPE‐PEG_2000_‐FA (2 µL, 100 mg mL^−1^) were added into a 3 mL chloroform/methanol (2:1, v/v) solution. Then rotated the mixture at 42°C for 10 min using a rotary evaporator and vacuum dried for 1 h. The resulting film was hydrated with 2 mL ddH_2_O, which contained CaCO_3_ (100 µL 200 mm), ICG (100 µL 2 mg mL^−1^), and 2 mg RhB. Then, the obtained solution was dispersed using ultrasound, dialyzed for 2 h, and centrifuged at 1200 rpm for 10 min to obtain PICL.

### 2.5 Characterization of CaCO_3_ and PICL

The morphology of CaCO3 and PICL were analyzed by transmission electron microscopy (TEM, TECNAI G2 F20, Philips, Netherlands). The characterization of CaCO_3_ and PICL was conducted in terms of particle size distribution, ζ potential, and morphology. A dynamic light scattering instrument (Zetasizer, Malvern, UK) was used to evaluate the hydrodynamic size, ζ potential, and drug stability. X‐ray photoelectron spectroscopy (ESCALAB 250XI) was used to perform XPS analysis on the CaCO_3_. The content of PTL was detected by high‐performance liquid chromatography (HPLC), and the content of ICG was detected by ultraviolet absorption spectroscopy. The entrapment efficiencies (EE) and the drug loading (DL) values were calculated as follows:

EE (%) = mass of a drug in the liposomes/mass of this drug loaded initially ×100.

DL (%) = mass of all drugs in the liposomes/mass of liposome ×100.

### 2.6 Detection of ROS generation

To evaluate the ability of PICL to produce ROS under US processing, DPBF was used to detect the production of ^1^O_2_. Add 15 µL DPBF solution (10 mm) to 3 mL ddH_2_O and irradiate with US (30 kHz, 0.3 W cm^−2^) for different durations (0, 2, 4, 6, 8, and 10 min). Record the UV visible absorption spectrum changes of DPBF at 420 nm. Add 15 µL DPBF solution (10 mm) to PICL aqueous solution (3 mL, ICG content 20 µg mL^−1^), using the same method to identify the ROS produced by ICG.

### 2.7 PH responsiveness detection of PICL

Detection of drug release using dialysis method in PBS buffer solutions with pH 5.5, 6.5, and 7.4. Then collect the samples at intervals of 0, 1, 3, 5, 7, 9, 12, and 24 h post‐addition. Detection of PICL particle size changes after processing at different time points. The content of PTL at each interval was detected by HPLC, the content of ICG was detected by ultraviolet absorption spectroscopy, and the calcium ion content at each interval was determined using a Calcium Colorimetric Assay Kit (Beyotime, China). And a graph depicting of the PTL, ICG, and calcium ion release ratio was constructed.

### 2.8 Cell uptake

Evaluating the efficiency of cell uptake of PICL by recording the fluorescence intensity of RhB within PICL. 4T1 cells and B16F10 cells were seeded into a 24‐well plate (1.0×10^5^ cells per well). 150 µL of RhB‐loaded PICL was added to 6 mL of complete culture medium, with 500 µL added to each well. The red fluorescence at 0, 1, 2, 4, 8 h post‐addition was observed using a confocal laser scanning microscope (CLSM, Nikon, A1‐ISTAR), and cell nuclei were stained with Hoechst 33342 (Solarbio, Beijing, China) prior to observation.

### 2.9 Biosafety verification of ultrasound

To evaluate the effects of different ultrasound treatment times and powers on cells, 4T1 cells were inoculated into 96‐well plates (1.0 × 10^4^ cells per well) and grew to be approximately 70–80% confluence before ultrasound treatment. Six ultrasound power gradients were set at 0, 0.1, 0.2, 0.3, 0.4, and 0.5 W cm^−2^, with each group treated for 3 min. The ultrasound duration gradient was set at 0, 1, 2, 3, 4, 5, 6, 7, 8, 9, and 10 min, each group at 0.2 W cm^−2^. After ultrasonic treatment, the cells were further incubated at 37°C for 6 h. The results were determined using cell counting kit‐8 (CCK‐8, Solarbio, Beijing, China) and EnSpire Multilabel Reader (PerkinElmer, Waltham, MA, USA).

### 2.10 Cytotoxicity assay of PICL

To assess the cytotoxicity of PICL, 4T1 cells, B16F10 cells, and HEK 293T cells were seeded in 96‐well plates, and allowed to grow to about 70–80% confluence, then PTL and PICL were added. Six gradients were set at 5, 10, 15, 20, 25, 30, and 50 µg mL^−1^ (equivalent PTL concentration of 2, 4, 6, 8, 10, 12, and 20 µm) with three replicates per group. The cells were incubated at 37°C for 24 h. At the 12th hour, the ultrasound group was subjected to ultrasound treatment (0.2 W cm^−2^, 3 min). After 24 h, each well was detected with CCK‐8 kit and EnSpire Multilabel Reader (PerkinElmer, Waltham, MA, USA).

### 2.11 Detection of intracellular ROS content

The capacity of PICL to generate ROS within cells was assessed using 2,7‐Dichlorodihydrofluorescein diacetate (DCFH‐DA, Solarbio, Beijing, China). 4T1 cells and B16F10 cells were cultured on 24‐well plates and incubated alongside the following groups: (1) control, (2) 8 µM PTL, (3) 100 µM CaCO_3_, (4) 20 µg mL^−1^ PICL, and (5) 20 µg mL^−1^ PICL+US, (B16F100 cells were treated with 10 µg mL^−1^ PICL). After 8 h, the PICL+US group underwent ultrasound treatment (0.2 W cm^−2^, 3 min). Following an additional 2 h of incubation, 4T1 cells and B16F10 cells were treated with DCFH‐DA and Hoechst 33342 for 20 min, and the fluorescence was observed using a laser scanning confocal microscope (LSCM).

### 2.12 Detection of intracellular Ca^2+^content

The intracellular Ca^2+^content was detected by Fluo‐3AM probe (Solarbio, Beijing, China) and Calcium Colorimetric Assay Kit (Beyotime, Shanghai, China). 4T1 cells were incubated with (1) control, (2) PTL, (3) CaCO_3_, (4) PICL, and (5) PICL+US when the cell reached 70–80% confluence. After 8 h, the PICL+US group was subjected to ultrasound treatment (0.2 W cm^−2^, 3 min). Following an additional 2 h of incubation, 4T1 cells were washed with D‐Hanks buffer and treated with the Fluo‐3AM probe at 37°C for 15 min. Then washed cells with HEPES buffer for three times, and each well was incubated with D‐Hanks buffer (containing 1% FBS) for 10 min. The fluorescence was observed with a laser scanning confocal microscopy (LSCM), and the changes in intracellular Ca^2+^content were detected by Calcium Colorimetric Assay Kit.

### 2.13 Calcein‐AM/PI cell staining experiment

4T1 cells and B16F10 cells were seeded on a 24‐well plate and incubated with (1) control, (2) PTL, (3) CaCO_3_, (4) PICL, and (5) PICL+US. After 8 h, the PICL+US group was subjected to ultrasound treatment (0.2 W cm^−2^, 3 min). Following an additional 2 h of incubation, the cells were treated with the Calcein‐AM/PI kit (Solarbio, Beijing, China), and incubated at 37°C for 20 min. Fluorescence was observed using a laser scanning confocal microscopy (LSCM).

### 2.14 Cell apoptosis measurement

To evaluate the ability of PICL to induce cell apoptosis, 4T1 cells were cultured on a 12‐well plate and incubated with (1) control, (2) PTL, (3) CaCO_3_, (4) PICL, and (5) PICL+US. After 8 h, the PICL+US group underwent ultrasound treatment (0.2 W cm^−2^, 3 min). Following an additional 2 h of incubation, the cells were treated with the Annexin V‐FITC Apoptosis Detection Kit (BD Bioscience, USA). Detect the distribution of cell populations in different quadrants using flow cytometry (FACSCalibur, BD, USA).

### 2.15 Mitochondrial membrane potential damage detection

4T1 cells were plated on a 12‐well plate and incubated with (1) control, (2) PTL, (3) CaCO_3_, (4) PICL, and (5) PICL+US. After 8 h, the PICL+US group was subjected to ultrasound treatment (0.2 W cm^−2^, 3 min). Following an additional 2 h of incubation, the cells were harvested and processed with the Mitochondrial Membrane Potential Kit (JC‐10 Assay, Solarbio, Beijing, China). Detection of cell population distribution in different quadrants using flow cytometry (FACSCalibur, BD, USA).

### 2.16 Bio‐TEM imaging of cells

Incubate 4T1 cells with control and PICL+US (20 µg mL^−1^, 0.2 W cm^−2^, 3 min) for 10 h. Collect cell precipitates and fix them with 2.5% glutaraldehyde at 4°C for 12 h. Then, the samples were subjected to biological transmission electron microscopy (bio‐TEM) analysis.

### 2.17 Detection of intracellular ATP content

Measure intracellular ATP content using the ATP Content Assay Kit (Solarbio, Beijing, China). 4T1 cells were seeded in 6‐well plates and incubated with (1) control, (2) PTL, (3) CaCO_3_, (4) PICL, and (5) PICL+US. After 8 h, ultrasonic treatment was performed (0.2 W cm^−2^, 3 min). After further incubation for 2 h, the cells were harvested. Extract intracellular ATP according to instructions and measure it using EnSpire Multilabel Reader.

### 2.18 Cell migration experiment

The in vitro metastatic potential of cancer was evaluated using a scratch wound healing assay. 4T1 cells were cultured in a 12‐well plate until they reached approximately 80% confluence. Use a sterile tip to draw straight‐line wound on the cell monolayer. Then the cells were incubated with (1) control, (2) PTL, (3) CaCO_3_, (4) PICL, and (5) PICL+US for 24 h. The wound was observed and photographed using an optical microscope at the start and after 24 h of cultivation. Calculate the scratch area using ImageJ software to quantify wound healing rate.

### 2.19 Sequencing and analysis of mRNA

4T1 cells were treated with control and PICL+US (20 µg mL^−1^, 0.2 W cm^−2^, 3 min). Following this, the cells were harvested and the total RNA was extracted utilizing TRIzol (9109, TaKaRa, Japan,). The high‐throughput sequencing of the transcriptome was carried out by Nanjin Personal Gene Technology Co., Ltd.

### 2.20 Measurement of intracellular Fe^2+^ content

Fluorescence imaging of Fe^2+^ in living cells was performed using the FerroOrange ferrous ion probe (F374, Dojindo, Japan). 4T1 cells were plated in 24‐well plates and incubated with (1) control, (2) PTL, (3) CaCO_3_, (4) PICL, and (5) PICL+US. After an additional 2 h, add FerroOrange working solution (1 µm) to the cells, and continue to cultivate for 30 min. The fluorescence was then observed under a laser scanning confocal microscopy (LSCM).

### 2.21 Detection of intracellular LPO and GSH content

The variations in intracellular LPO and GSH content were assessed using a LPO Content Detection Kit (Solarbio, Beijing, China) and Reduced Glutathione (GSH) Detection Kit (Solarbio, Beijing, China). 4T1 cells were plated in a 12‐well plate and incubated with (1) control, (2) PTL, (3) CaCO_3_, (4) PICL, and (5) PICL+US. Following 8 h, an ultrasound treatment was administered (0.2 W cm^−2^, 3 min). After an additional 2 h, the cells were harvested via trypsin digestion. The cells were then processed using the assay kit, and the absorbance values were detected using EnSpire Multilabel Reader.

### 2.22 qRT‐PCR assay

After treating 4T1 cells with low and high concentrations of PICL (10 and 20 µg mL^−1^), total RNA was extracted using TRIzol and reversed transcribed into cDNA by Hifair III 1st Strand cDNA Synthesis SuperMix for qPCR (Yeasen, Shanghai, China). The relative qPCR was performed with Hieff qPCR SYBR Green Master Mix (Low Rox Plus) (Yeasen, Shanghai, China). Applied Biosystems 7500 was used for numerical determination, and the thermal cycling program consisted of 5 min at 95°C followed by 45 cycles at 95°C for 10 s, 57°C for 20 s, and 72°C for 34 s. All primers for qRT‐PCR are listed in Table S1, Supporting Information.

### 2.23 Western blot assay

After low and high concentrations (10 and 20 µg mL^−1^) of PICL treatment, 4T1 cells were lysed using RIPA cell lysate to extract total cell proteins. After denaturation at 99°C, the protein was electrophoretic on 10 % SDS polyacrylamide gel (80 U, 30 min, 130 U, 55 min) and transferred to NC membrane (80 U, 2 h). Seal the membrane in 5 % (w/v) skimmed milk at 37°C for 1 h. Dilute the primary antibody to 1:1000 and incubate overnight at 4°C. Dilute the secondary antibody with 1:20,000 and incubate at 37°C for 1 h. After washing, use Super ECL Detection Reagent (Yeasen, Shanghai, China) to develop and print, and visualize in the Chemiluminescence multicolor fluorescent gel imaging system.

### 2.24 In vivo anti‐tumor immunotherapy

Take female Balb/c mice (6–8 weeks old) and inject subcutaneously 100 µL 4T1 cells were dispersed in 1640 serum‐free medium to induce tumors (2.0 × 106 cells injections per mouse). When the tumor volume reached about 100 mm^3^, the tumor‐bearing mice are randomly divided into 6 groups (*n* = 5): (1) control, (2) US, (3) PTL (1 mg mL^−1^), (4) CaCO_3_ (1.5 mg mL^−1^), (5) PICL (3 mg mL^−1^), and (6) PICL+US. Inject 20 mg kg^−1^ into each mouse through the tail vein. Inject once every 2 days, a total of three times, and give ultrasound treatment (1 MHz, 0.5 W cm^−2^, 4 min) to the mice of US and PICL+US group 24 h after each injection. Monitor the tumor volume and the mouse weight during treatment. At the end of treatment (the 16th day), euthanize the mice. Remove the tumor and perform histological staining, including hematoxylin‐eosin (H&E), terminal deoxynucleotidyl transferase dUTP nick end labeling assay (TUNEL), and Ki‐67 immunohistochemical staining, GPX4 and ACSL4 to examine the anticancer effect.

### 2.25 In vivo biosafety evaluation of PICL

Mice were administered an intravenous injection of 150 µL of PICL. In vivo multispectral imaging was used to observe the drug metabolism at 2, 8, 24, and 48 h post‐injection. Following the treatment, blood samples from mice were collected and centrifuged to obtain serum for blood biochemical testing to analyze changes in alanine aminotransferase (ALT), aspartate aminotransferase (AST), creatine kinase (CK), blood urea nitrogen (UREA), creatinine (CREA) and lactate dehydrogenase (LDH) contents. Randomly select three mice from each group to collect serum samples. Simultaneously collect the main organs of mice (heart, liver, spleen, lungs, and kidneys) for H&E staining to evaluate the biosafety of PICL.

### 2.26 Detection of DC maturation and cytotoxic T lymphocyte (CTL) activation

The maturation rate of dendritic cells (DC) in mouse tumors and lymph nodes was examined. Cells were co‐stained with FITC anti‐mouse CD11c, APC anti‐mouse CD80 antibodies, PE anti‐mouse CD86 and analyzed by flow cytometry. T cell activation was analyzed in the tumors and spleens of the mice. Spleen and tumor cell suspensions were stained with FITC anti‐mouse CD3, PE anti‐mouse CD8a, APC anti‐mouse CD4 and PE/Cyanine7 anti‐mouse CD45 antibody and analyzed by flow cytometry. All the above antibodies were from BioLegend. Randomly select three mice from each group to collect tumors, spleens, and lymph nodes. Three biological replicates were conducted for each combination.

### 2.27 Immune factor detection

Mouse serum was collected, and changes in TNF‐α, IL‐2, and IL‐12 in the mouse blood were detected using mouse TNF‐α ELISA kit, mouse IL‐2 ELISA kit, and mouse IL‐12 ELISA kit. The above ELISA kits were from Solorbio. Three biological replicates were conducted for each combination.

### 2.28 Statistical analysis

Quantitative data is represented as mean ± SD and analyzed by Origin 2021 or GraphPad Prism 8.0. Compare multiple groups using one‐way analysis of variance (ANOVA). The statistical significance is denoted as *P < 0.05, **P < 0.01, ***P < 0.001.

## 3 RESULTS AND DISCUSSION

### 3.1 Synthesis and characterization of PICL nanomaterials

**FIGURE 1:**
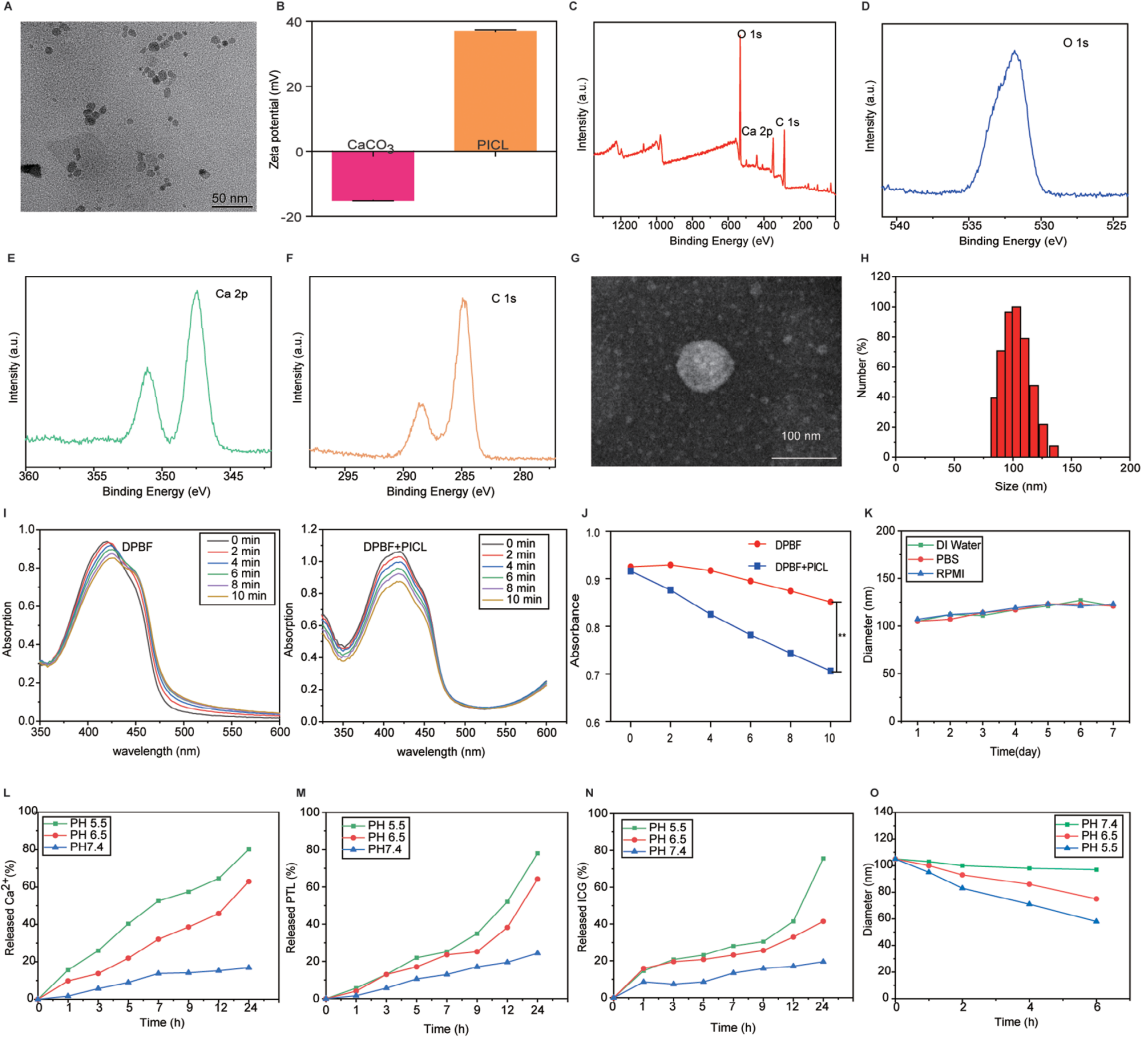
Characterization of PICL Nanomaterials. (A) TEM image of CaCO_3 _NPs. (B) TEM image of PICL NPs. (C) Zeta potentials of CaCO_3_ and PICL NPs. (D) XPS full survey spectra of CaCO_3_. (E–G) C 1s, Ca 2p and O 1s peaks XPS spectra of CaCO_3_. (H) Hydrodynamic size of PICL NPs. (I) The UV visible absorption spectra and (J) statistical analysis of 424 nm absorption peak of DPBF probes under different treatments. (K) The hydrodynamic diameter of PICL in deionized water, PBS, and RPMI (containing 10% serum) at 4°C for 7 days. Percentage of (L) Ca^2+^, (M) PTL, and (N) ICG released by PICL at different time points under different pH. (O) Particle size changes of PICL at different time points under different pH.

The preparation process of PICL is illustrated in Scheme 1A. Firstly, the morphology of CaCO_3_ nanoparticles was characterized by TEM, revealing a uniform spherical structure with particle size of around 18 nm (Figure 1A), and ζ potential of −18.9 mV (Figure 1B). X‐ray photoelectron spectroscopy (XPS) analysis clearly indicate the presence of O, Ca, and C elements in CaCO_3_, indicating the successful preparation of CaCO_3_ (Figure 1C–F). Subsequently, PICL nanoparticles loaded with CaCO_3_ nanoparticles, PTL, and ICG were synthesized using the thin film hydration method. The morphology of PICL was confirmed to be a uniform spherical structure by TEM (Figure 1G). The particle size and ζ potential of PICL were determined to be 103 nm and 43.2 mV, respectively (Figure 1B,H). The encapsulation efficiency of PTL was calculated to be 83.5% through HPLC analysis (Figure S1, Supporting Information), while the encapsulation efficiency of ICG measured by UV spectrophotometer is 97.5% (Figure S2, Supporting Information). After calculation, the drug loading is 72.5%.

The acoustic dynamic performance of PICL was evaluated using a DPBF probe. DPBF is a fluorescent probe indicating ^1^O_2_ with high specificity. Upon interaction with ^1^O_2_, DPBF undergoes irreversible oxidation, leading to a rapid decrease in absorbance intensity at ultraviolet‐visible light. As depicted in Figure 1I. In the presence of PICL, the characteristic absorption peak of DPBF decreased with increasing US irradiation time, whereas the absorbance change of the control group (DPBF+US) under US irradiation was negligible. The UV absorption peak at 425 nm was selected for data statistical analysis, and the results showed significant differences between the two groups (Figure 1J). This indicates that US stimulation can trigger the production of ^1^O_2_ by PICL, confirming the acoustic dynamic effect of PICL. Furthermore, no significant changes were found in the particle size of PICL when stored at 4°C for 1 week in double distilled water, PBS buffer, and serum containing medium (Figure 1K). Next, the response ability of PICL to different pH levels was tested. The results indicate that the highest drug release can be achieved at pH 5.5. Of course, at pH 6.5, PICL can still effectively release 64.23% PTL, 41.46% ICG, and 62.94% calcium ions, indicating that PICL can also achieve effective response and drug release in the tumor microenvironment (Figure 1L–N). In addition, we also detected particle size changes in PICL treated with different pH levels. It was found that the particle size of PICL was relatively stable at pH 7.4, and both pH 6.5 and pH 5.5 had an impact on the particle size of PICL. Revealing that the structure of PICL is affected under slightly acidic pH conditions (Figure 1O).

### 3.2 PICL induces cell apoptosis by producing ROS and calcium overload

The uptake of PICL by tumor cells was assessed using RhB‐labeled PICL. Incubate PICL with 4T1 and B16F10 cells for different durations (0, 1, 2, 4, and 8 h). As shown in Figures S3 and S4, Supporting Information, the red fluorescence intensity inside the cells significantly increased with prolonged incubation time, confirming the effective endocytosis of PICL by the cells.

**FIGURE 2:**
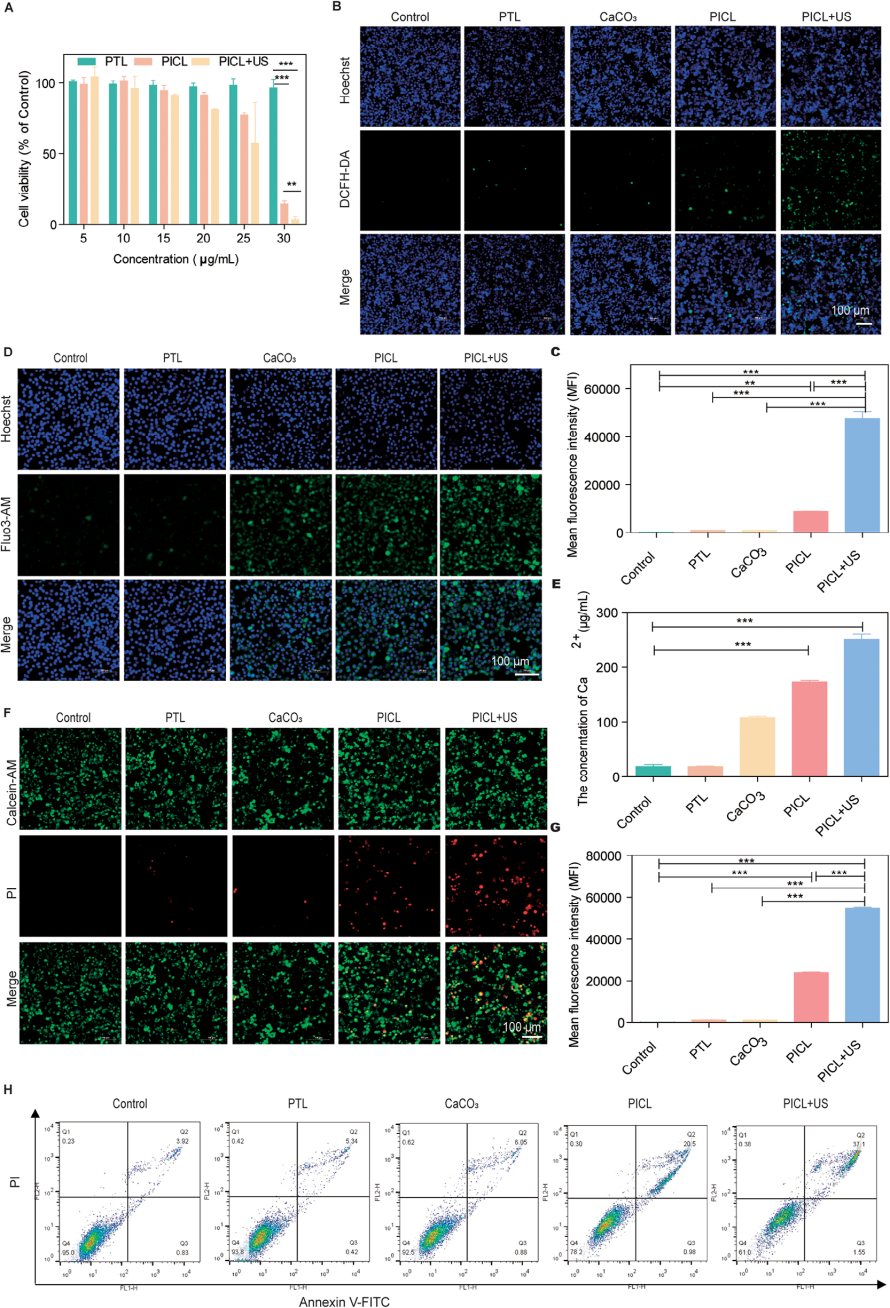
PICL induces cell apoptosis by producing ROS and calcium overload. (A) Cell viability of 4T1 cells incubated with different concentrations. *n* = 3. (B) CLSM images and (C) Mean fluorescence intensity of 4T1 cells stained with DCFH‐DA after different treatments. Scale bar =100 µm. (D) CLSM image of intracellular Ca^2+^ stained by Fluo3‐AM. Scale bar =100 µm. (E) The Calcium Colorimetric Assay Kit detects the intracellular Ca^2+^ content after different treatments. *n* = 3. (F) CLSM images and (G) Mean fluorescence intensity of 4T1 cells stained with calcein‐AM (green) and PI (red) after different treatments. Scale bar =100 µm. (H) Flow cytometry analysis of 4T1 cells stained with membrane associated protein FITC and PI after various treatments. *P*‐values: *P < 0.05, **P < 0.01, and ***P < 0.001.

The cytotoxicity of PICL was investigated by assessing cell viability using the CCK‐8 kit. Initially, the safety of ultrasound for 4T1 cells was confirmed. The viability of 4T1 cells was unaffected by ultrasound at a power of less than 0.3 W cm^−2^ for 3 min, and by ultrasound at a power of 0.2 W cm^−2^ for 10 min (Figure S5A,B, Supporting Information). Therefore, it was deemed safe to use ultrasound at 0.2 W cm^−2^ for 3 min in subsequent cell experiments. Next, the cytotoxicity of PICL was evaluated using 4T1 cells, B16F10 cells and HEK 293T cells. At a PICL concentration of 10 µg mL^−1^, its cytotoxicity to 4T1 cells was negligible without ultrasound irradiation. When the concentration exceeded 15 µg mL^−1^, PICL began to exhibit significant cytotoxicity in a dose‐dependent manner (Figure 2A). Moreover, US treatment increased the ROS production of PICL, resulting in stronger toxicity to cells. Therefore, the cytotoxicity of PICL+US treatment was significantly enhanced. Consistent results were also obtained in B16F10 cells, with even lower effective doses of PICL (Figure S6A, Supporting Information). In addition, the toxicity of PICL to healthy cells was studied in HEK 293T cells, and it was found that within the same PICL dose range, no significant cytotoxicity was observed on HEK 293T cells, even at a dose of 50 µg mL^−1^ (Figure S6B, Supporting Information).

To verify whether ultrasound could enhance the ROS production rate of PICL within cells, we used the DCFH‐DA fluorescent probe to detect the ROS production capacity of PICL in 4T1 cells and B16F10 cells (Figure 2B and Figure S7, Supporting Information). The results showed that only sporadic green fluorescence was observed in the control, PTL, or CaCO_3_ groups. Without ultrasound treatment, weak green fluorescence was observed in the PICL group, possibly due to increased oxidative stress levels in the cells caused by PTL release. However, after adding US, significant green fluorescence was observed, and the mean fluorescence intensity statistics also showed that the PICL+US group had a much higher value the other groups (Figure 2C), indicating that the PICL+US group could generate a large amount of ROS within cells.

To verify whether PICL could increase the calcium content within cells, we used the Fluo3‐AM probe to observe changes in Ca^2+^ content within 4T1 cells (Figure 2D). Compared with the control group and PTL group, 4T1 cells in the CaCO_3_ treatment group displayed stronger green fluorescence. However, more significant green fluorescence was observed in the PICL and PICL+US groups. This is because liposome encapsulation improved the efficiency of cell absorption of CaCO_3_. In addition, the intracellular calcium ion content was quantitatively detected using a calcium colorimetric assay kit (Figure 2E), yielding consistent results.

To evaluate the in vitro anti‐tumor effect of PICL, AM/PI staining and flow cytometry were employed. Figure 2F and Figure S8, Supporting Information display the co‐staining results of calcein AM and PI in 4T1 cells and B16F10 cells. The control group, PTL group, and CaCO_3_ group all displayed green fluorescence, indicating that single treatments had minimal damage to the cells. In the PICL group, a small number of red fluorescence was observed, while in the PICL+US group, many cells displayed red fluorescence, indicating significant cytotoxicity. The fluorescence intensity statistics further confirmed this result (Figure 2G). Subsequently, the Annexin V‐FITC cell apoptosis detection kit was used to further quantify cell apoptosis. As shown in Figure 2H, the live cells ratios of the control group, PTL group, and CaCO_3_ group all exceeded 92%, indicating that the impact on cell viability was negligible. The proportion of late apoptotic cells in the PICL group was 20.5%, while the apoptosis rate in the PICL+US group was the highest, at 37.1%, indicating that PICL exerts anti‐tumor effects by inducing tumor cell apoptosis through increasing intracellular calcium ion content and producing a large amount of ROS.

### 3.3 PICL damages mitochondria and inhibits 4T1 cell migration

**FIGURE 3:**
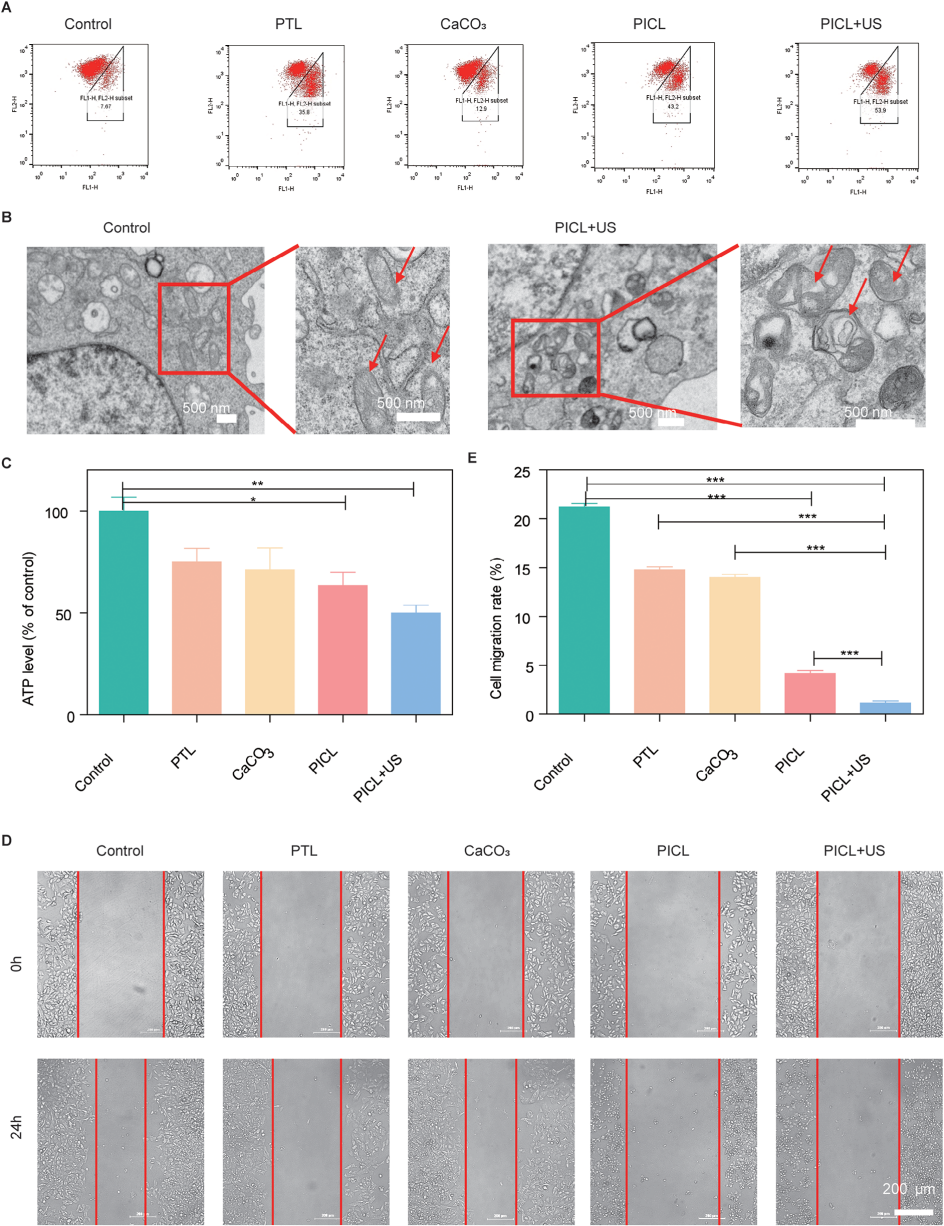
(A) Flow cytometry was used to analyze the decrease in mitochondrial membrane potential of 4T1 cells stained with JC‐10 after different treatments. (B) Biological transmission electron microscopy images of 4T1 cells before and after PICL+US treatment. Use red arrows to indicate mitochondria. (C) The ATP content assay kit detects changes in intracellular ATP content after different treatments. *n* = 3. (D) Cell images from scratch wound healing experiments at 0 and 24 h. Scale bar =100 µm. (E) The results of cell migration rate. *P*‐values: *P < 0.05, **P < 0.01, and ***P < 0.001.

Mitochondria serve as the power sources for eukaryotic cells, contributing to energy production, calcium regulation, ROS generation, and metabolic signal transduction^[^ ^34^, ^35^, ^36^, ^37^ ^]^. They play a pivotal role in maintaining cellular homeostasis and survival^[^ ^38^ ^]^. Roughly 90% of intracellular ROS is derived from mitochondria^[^ ^39^ ^]^, but an excessive accumulation of ROS can trigger mitochondrial oxidative damage^[^ ^40^ ^]^. Concurrently, an overload of intracellular Ca^2+^ that elevates the mitochondrial Ca^2+^ concentration can lead to issues such as a decrease in mitochondrial membrane potential, a reduction in ATP levels, alterations in mitochondrial morphology, and mitochondrial respiratory disorders^[^ ^41^ ^]^. To examine the impact of PICL on intracellular mitochondria, we first utilized the JC‐10 Assay in conjunction with flow cytometry to detect changes in the mitochondrial membrane potential following 4T1 cell treatment. As depicted in Figure 3A, the PICL group led to a 43.2% decline in mitochondrial membrane potential, suggesting that Ca^2+^ overload and excessive ROS accumulation can inflict mitochondrial damage. The mitochondrial membrane potential in 4T1 cells treated with PICL+US decreased by 57.9%, indicating that an increase in intracellular ROS can intensify mitochondrial damage. To further visually assess the extent of mitochondrial damage, we employed a bio‐TEM) to observe changes in mitochondrial morphology within 4T1 cells. The results revealed that, compared to the control group, mitochondria were noticeably swollen and the cristae were flattened following PICL+US treatment (Figure 3B).

To confirm whether mitochondrial damage influences cell function, we initially used an ATP Content Assay Kit to detect changes in intracellular ATP content. The results demonstrated that the intracellular ATP content in the PICL+US group significantly decreased, amounting to only 50% of that in the control group (Figure 3C). This suggests that the cellular energy supply function of mitochondria was notably inhibited following PICL+US treatment. Given that tumor cell migration requires substantial ATP energy, we subsequently tested the migration capability of 4T1 cells using a scratch wound healing experiment. The results indicated the slowest reduction in scratch spacing in the PICL+US group, with a cell migration rate of 1% (Figure 3D,E). This suggests that PICL+US effectively curtailed the migration ability of 4T1 cells, implying a potential preventive effect on tumor metastasis.

### 3.4 PICL achieves anti‐tumor immunity through ferroptosis pathway

**FIGURE 4:**
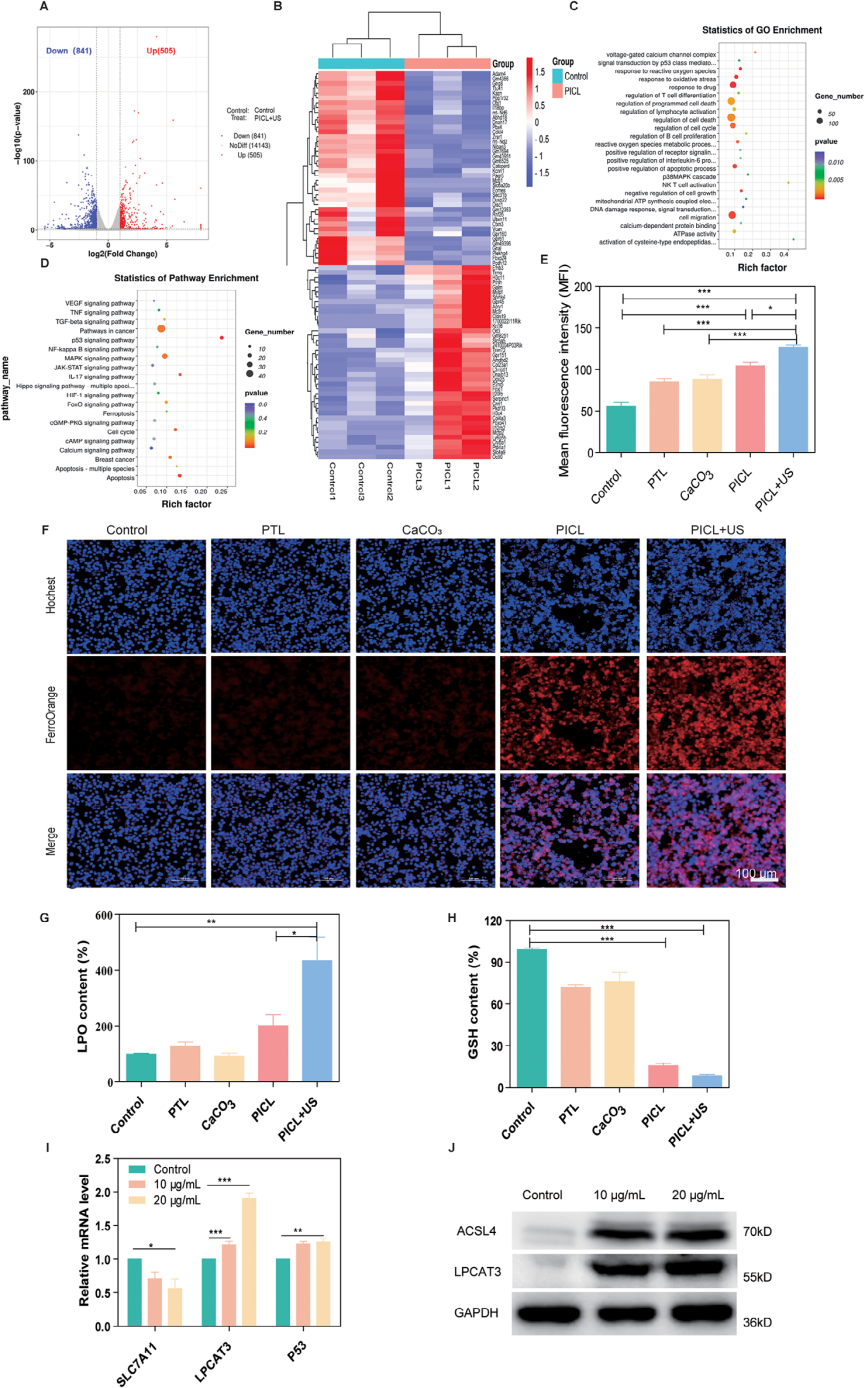
RNA‐seq analysis to explore the biological mechanism of PICL+US anti‐tumor effect. (A) Volcano map of differentially expressed genes in the control group and PICL+US group. Genes with a 2‐fold change and a *P* value of <0.05 are highlighted in blue and red, indicating downregulated and upregulated genes, respectively. (B) GO enrichment based on RNA seq after PICL+US treatment. (C) Cluster heat map of differentially expressed genes after PICL+US treatment. (D) Bubble diagram of differentially expressed genes enriched in the Kyoto Encyclopedia of Genes and Genomes pathway. (E) Mean fluorescence intensity and (F) CLSM images of 4T1 cells stained with FerroOrange fluorescence probe after treated with different formulas. Scale bar =100 µm. (G) The lipid peroxide (LPO) content detection kit detects the intracellular LPO content after different treatments. n = 3. (H) The Reduced Glutathione Detection Kit Calcium Colorimetric Assay Kit detects the reduced GSH content in 4T1 cells after different treatments. *n* = 3. (I) The results of qRT‐PCR to detect the expression of SLCl7A11, P53 and LPCAT3 in 4T1 cells treated with different concentrations of PICL (10 µg mL^−1^ and 20 µg mL^−1^). (J) The results of Western blot to detect the expression of ACSL4 and LPCAT3 in 4T1 cells treated with different concentrations of PICL (10 and 20 µg mL^−1^). *P*‐values: *P < 0.05, **P < 0.01, and ***P < 0.001.

To elucidate the potential molecular mechanism of PICL+US on tumor cells, we conducted an RNA‐seq analysis on 4T1 cells. Three biological replicates were conducted for each combination. The results revealed that in the PICL+US treatment group, a total of 1346 genes were regulated compared to the control group. Among them, 505 genes were induced, and 841 genes were suppressed (Figure 4A, Table S2, Supporting Information). We selected the 80 most significantly differentially expressed genes to create a heatmap, as shown in Figure 4B. Subsequently, to explore specific biological processes, we utilized gene ontology (GO) to analyze gene set enrichment (Figure 4C). GO analysis enriched genes whose expression changed in biological processes such as cell apoptosis, cell migration, cell oxidative stress response, and cell response to calcium ions, which aligns with previous results. To further identify the immune pathways involved in the induction, we performed Kyoto Encyclopedia of Genes and Genomes (KEGG) analysis. KEGG analysis enriched genes whose expression changed in related pathways such as P53 signaling pathway, NF‐κB signaling pathway, MAPK signaling pathway, calcium signaling pathway, FoxO signaling, IL‐17 signaling pathway, Hippo signaling etc. (Figure 4D). Notably, we observed that the ferroptosis pathway was enriched, prompting us to design subsequent studies to further explore the role of the ferroptosis pathway.

Ferroptosis is primarily characterized by iron accumulation, ROS accumulation, and lipid peroxidation. In our previous experiments, we have already detected changes in intracellular ROS content. Therefore, we proceeded to investigate the status of intracellular iron ions and lipid peroxidation to determine whether PICL could induce ferroptosis. Initially, we employed the FerroOrange probe to examine the content of Fe^2+^ within cells. As depicted in Figure 4E,F, the red fluorescence in the PICL+US group significantly increased compared to the control, PTL, and CaCO_3_ groups, suggesting that PICL+US can enhance Fe^2+^ accumulation in 4T1 cells. We further utilized a lipid peroxide (LPO) Content Detection Kit to investigate the intracellular LPO content. We discovered that the intracellular LPO content in the PICL+US group was 4.34 times that of the control group, indicating a significant increase compared to other groups (Figure 4G). This suggests that PICL+US can promote the accumulation of lipid peroxidation products within 4T1 cells. Lastly, we used a Reduced Glutathione Detection Kit to study the intracellular GSH level. We found that the intracellular GSH in the PICL+US group was significantly depleted, amounting to only 8.33% of the control group (Figure 4H). This indicates that PICL+US can effectively promote the consumption of reduced GSH. These results collectively suggest that PICL+US can significantly induce 4T1 cells ferroptosis.

Based on the results of differential gene enrichment, we noticed that the expression of the SLC7A11 gene was downregulated. SLC7A11 is one of the subunits of system Xc^−^, which regulates ferroptosis by transporting extracellular cystine^[^ ^42^ ^]^. Inhibition of SLC7A11 leads to the inactivation of cysteine‐dependent glutathione peroxidase, which can enhance intracellular lipid peroxidation and ferroptosis^[^ ^43^, ^44^ ^]^. Therefore, we chose the SLC7A11 gene to verify its expression changes by qRT‐PCR, and the results found that after PICL+US treatment, the expression of SLC7A11 was indeed reduced, indicating that PICL+US inhibited the expression of SLC7A11. In addition, recent studies have found that SLC7A11 is one of the key targets of P53, which is negatively regulated by P53 protein^[^ ^16^, ^45^ ^]^. By detecting the expression of the P53 gene by qRT‐PCR, it was found that after PICL+US treatment, the expression of p53 was upregulated (Figure 4I), indicating that PICL+US may regulate tumor cell ferroptosis through the P53‐ SLC7A11 pathway. Subsequently, the expression changes of ferroptosis‐related proteins ACSL4 and LPCAT3 were detected by Western blot, and the results showed that after PICL+US treatment, the expression of ACSL4 and LPCAT3 proteins increased (Figure 4J), and the results of qRT‐PCR also showed that the expression of LPCAT3 increased (Figure 4I), indicating that PICL+US may regulate ferroptosis through the ACSL4/LPCAT3 pathway. Therefore, PICL may regulate cell ferroptosis by regulating related signaling pathways of ferroptosis, thereby exerting anti‐tumor immunity.

### 3.5 Evaluation of the in vivo anti‐tumor effect of PICL

**FIGURE 5:**
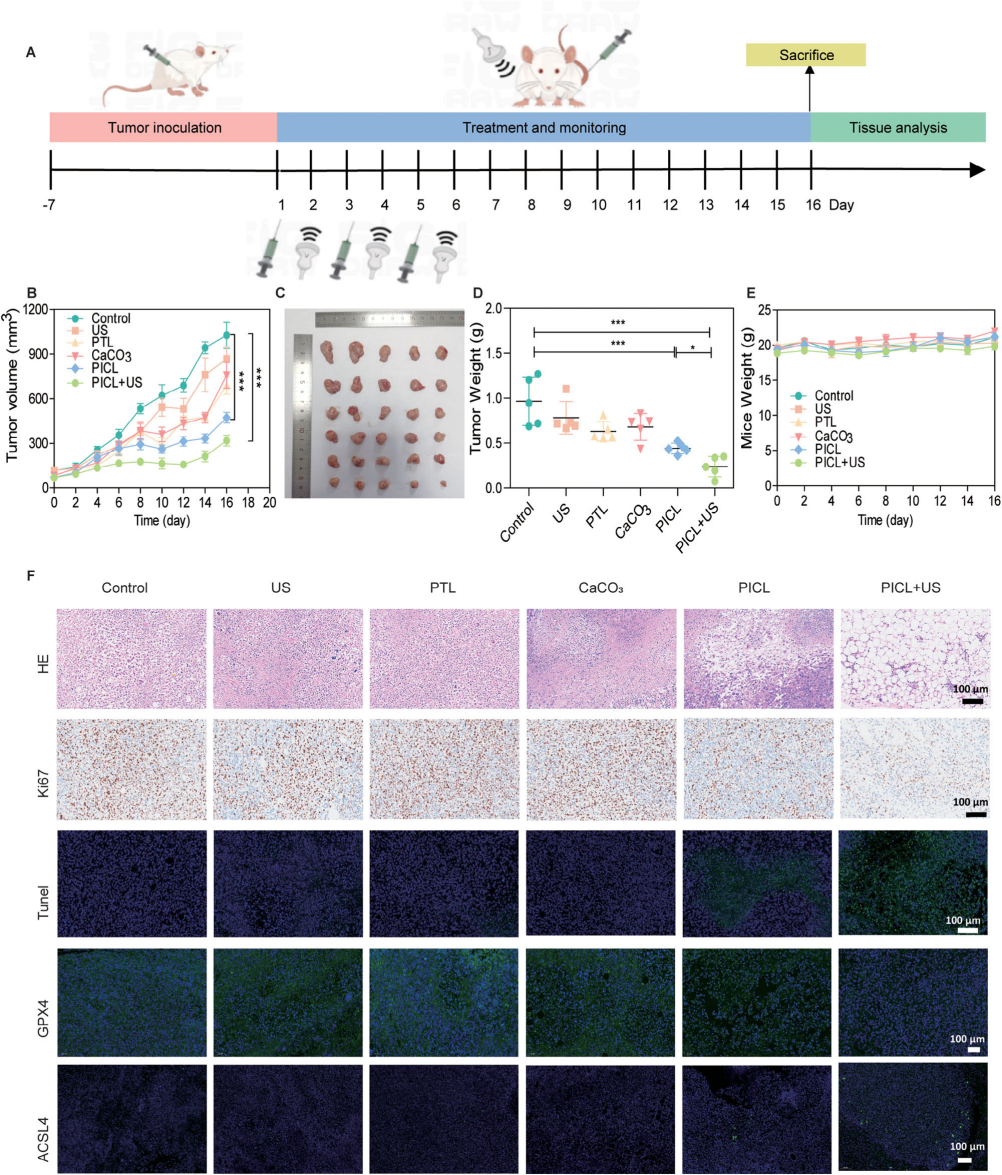
Evaluation of the in vivo anti‐tumor effect of PICL. (A) Schematic diagram of in vivo treatment plan. (B) The tumor growth curves of 4T1 tumor bearing mice after different treatments. (C) Tumor photos obtained from different groups of tumor‐bearing mice after a 16‐day treatment period (*n* = 5 in each group). (D) Statistical charts of tumor weight obtained from different groups of tumor‐bearing mice after a 16‐day treatment period (*n* = 5 for each group). (E) Line chart of weight changes in different groups of tumor‐bearing mice during the 16‐day treatment period. (F) Histological images of tumor sections collected from tumor bearing mice stained with H&E, antigen Ki‐67, TUNEL, GPX4, and ACSL4 antibodies. *P*‐values: *P < 0.05, **P < 0.01, and ***P < 0.001.

We further investigated the anti‐tumor activity of PICL in BALB/c mice bearing 4T1 tumors. As depicted in Figure 5A, 4T1 cells were subcutaneously implanted into the right axillary region of the BALB/c mice. Observe the tumor volume and when the average tumor volume reached approximately 100 mm^3^, randomly divide the tumor‐bearing mice into six groups (*n* = 5) for treatment. Over a 16‐day observation period, the tumor volume change curve of mice showed that the tumor volumes for the control and US groups continued to increase (Figure 5B and Figure S9, Supporting Information). And there was no significant difference in tumor growth between the two groups, suggesting that the impact of US exposure on tumor suppression is negligible. The PTL and CaCO_3_ groups exhibited a little inhibitory effect on tumor growth, possibly due to mild oxidative stress and Ca^2+^ overload causing tumor cell damage. Moreover, in the PICL group, we observed a relatively effective suppression of tumor growth, with the tumor volume increasing slowly. This suggests that PICL, to some extent, inhibits tumor growth by inducing apoptosis and ferroptosis in tumor cells. Compared to other groups, the tumor growth in the PICL+US treatment group significantly inhibited, which can be attributed to the enhancement of oxidative stress effects. After treatment, the tumor was removed, weighed, and photographed, with results showing a positive correlation with the tumor volume of the mice (Figure 5C,D). Notably, under different treatments, the weight of the tumor‐bearing mice steadily increased (Figure 5E), indicating that the adverse effects of this treatment on mouse metabolism can be disregarded.

At the end of treatment, collect the main organs and tumors of the mice for histological examination to evaluate the therapeutic effect. H&E staining of the major organs in each group did not reveal any organ damage, confirming the biocompatibility and good tolerance of PICL+US irradiation treatment (Figure S10, Supporting Information). We also performed blood biochemistry analysis on mice blood to reflect liver and kidney function (Figure S11, Supporting Information). Compared to the saline group, there was no significant difference in the liver and kidney function indicators of the PICL+US group, indicating that it did not induce hematological, liver, or kidney toxicity. This also attests to the safety of the treatment. Imaging results showed that a large amount of PICL accumulates at the tumor site within 24 h and was mostly metabolized within 48 h (Figure S12, Supporting Information). Subsequently, tumor tissue was analyzed using H&E, TUNEL and Ki‐67 immunohistochemical staining to further evaluate the treatment effects (Figure 5F). H&E staining showed that, compared to the control group, the tumor tissue of the PICL+US group displayed large cavities and a significant amount of spilled cytoplasm. TUNEL staining images showed that the tumors in the PICL+US group exhibited the most significant cell necrosis and damage. The immunohistochemical results of Ki‐67 showed a significant decrease in the positive rate in this group, further indicating its significant inhibitory effect on tumor cell proliferation. Additionally, we used GPX4 and ACSL4 antibodies for tissue staining to detect the expression of GPX4 and ACSL4 in tumor tissue, verifying the induction of ferroptosis in tumors by PICL (Figure 5F and Figure S13, Supporting Information). The results showed that, compared to other groups, PICL treatment resulted in a significant decrease in GPX4 expression in tumors and a significant increase in ACSL4 expression. Indicating that PICL can also induce tumor ferroptosis in tumor tissue.

### 3.6 In vivo immune activation effect of PICL

**FIGURE 6:**
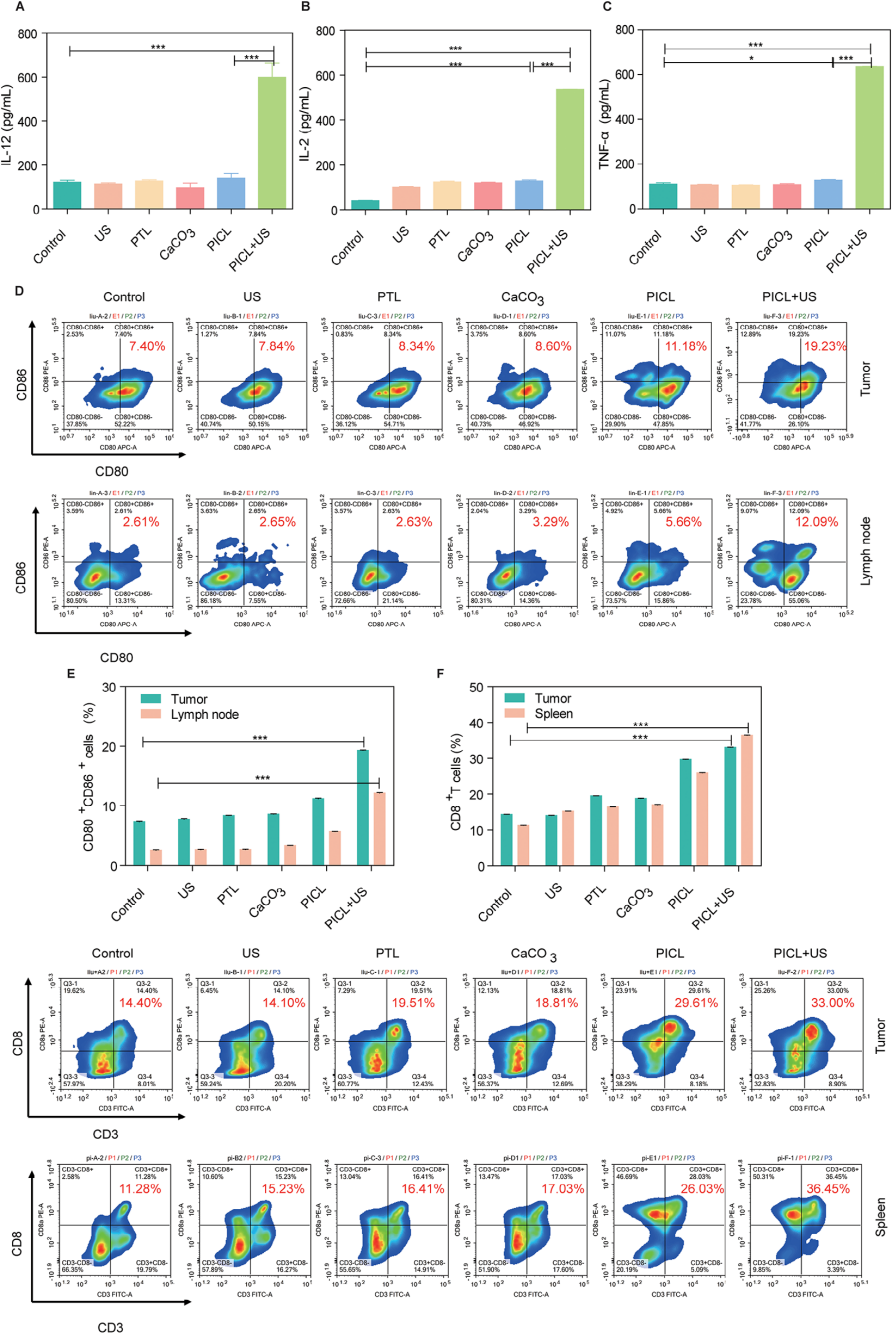
In vivo immune activation effect of PICL. The secretion levels of (A) IL‐12, (B) IL‐2, and (C) TNF‐α in the serum of tumor‐bearing mice after different treatments (*n* = 3). (D) Flow cytometry analysis and (E) quantitative statistical analysis of DC cell maturation in tumor and tumor draining lymph nodes of tumor‐bearing mice after different treatments (*n* = 3). (F) Flow cytometry analysis and (G) quantitative statistical analysis of CD8^+^T cells in the tumor and spleen of tumor‐bearing mice after different treatments (*n* = 3). *P*‐values: *P < 0.05, **P < 0.01, and ***P < 0.001).

To further explore whether nanoparticles can activate immune responses in vivo, we first detected the levels of immune related cytokines in serum through ELISA. Cytokines play a crucial role in the development and differentiation of immune cells. As shown in Figure 6A–C, three immune related cytokines, interleukin‐12 (IL‐12), interleukin‐2 (IL‐2), and tumor necrosis factor‐α (TNF‐α) were observed in the PICL+US group The serum levels were 4.96 times, 13.25 times, and 5.83 times higher than the control group, respectively, effectively promoting the expression of immune factors in mice. Then, we studied the ability of PICL to activate DC maturation, CD8^+^T,‐ and CD4^+^T cell activation by collecting tumor, spleen, and tumor draining lymph nodes using flow cytometry to evaluate immune activation in mice. The proportion of CD80^+^CD86^+^cells in tumor tissue is approximately 2.6 times higher in the PICL+US group (19.23%) than in the control group (7.4%). The PICL+US group (12.09%) in the draining lymph nodes was approximately 4.7 times higher than the control group (2.61%) (Figure 6D,E). This indicates that PICL+US significantly promotes the maturation of DCs and activates anti‐tumor immune responses. In addition, the CD8^+^T and CD4^+^T cells in the PICL+US group were significantly elevated. Among them, the content of CD8^+^T and CD4^+^T cells in the tumor was 2.3 times and 2.1 times higher than that of the control group, and the spleen was 3.2 times and 1.7 times higher than the control group, respectively (Figure 6F,G and Figure S14A,B, Supporting Information). This indicates that PICL+US can recruit more CD8^+^T and CD4^+^T cells to promote tumor infiltration of T lymphocytes.

## 4 CONCLUSION

To summarize, a nanoparticle formulation, PTL/ICG‐CaCO_3_@Lip, anchored on liposomes, has been ingeniously crafted to amplify ferroptosis in tumor cells, serving as a promising avenue for cancer immunotherapy. This nanoparticle substantially bolsters ROS levels within tumor cells, invoking oxidative stress that results in mitochondrial compromise and the subsequent impairment of cellular functions. RNA‐seq analysis insights corroborate that PTL/ICG‐CaCO_3_@Lip efficiently propels ferroptosis in tumor cells by regulating P53 to downregulate the SLC7A11 protein, inhibiting the system Xc^−^, and triggering the ACSL4/LPCAT3 pathway. Moreover, the PTL/ICG‐CaCO_3_@Lip‐driven ferroptosis immunotherapy not only curtails tumor progression but also ignites the murine immune response. Intriguingly, this study showcases the ability of PTL/ICG‐CaCO_3_@Lip to thwart tumor cell migration, hinting at its potential role in mitigating tumor metastasis, and thereby offering a groundbreaking approach for the next frontier in cancer immunotherapy.

## AUTHOR CONTRIBUTIONS

Jun Kang and Jin Chang—conceived and designed the project; Xue Bai and Silong Wei—performed the experiments and analysis; Yangsui Liu and Yun Wang—drew schematic on manuscript; Huansong Li and Xi Yang—provided technical input on this project. Xue Bai, Bo Yuan, and Jun Yan—wrote the manuscript. All authors read and approved the final manuscript.

## CONFLICT OF INTEREST STATEMENT

The authors declare no conflicts of interest.

## Supporting information



Supporting information

Supporting information

## Data Availability

The data that support the findings of this study are available from the corresponding author upon reasonable request.
