# Supplementary material for: A pH responsive nanocomposite for combination sonodynamic‐immunotherapy with ferroptosis and calcium ion overload via SLC7A11/ACSL4/LPCAT3 pathway
Source: Exploration (Beijing). 2024 Jun 26;5(1):20240002. doi: 10.1002/EXP.20240002 (PMC11875445; doi:10.1002/EXP.20240002)
Supplement: Supplementary file 1 — Supporting information [file EXP2-5-20240002-s002.docx]

*Supporting Information*

Xue Bai^a, b, 1^, Jun Kang^a, b, c, 1, *^，Silong Wei^d, 1^, Yun Wang^e, 1^, Yangsui Liu^e^，Bo Yuan^e^，Qian Lu^e, f, g^, Huansong Li^e, *^, Jun Yan^e, f, g, *^, Xi Yang^h, *^, Jin Chang^a, b, *^

a. School of Life Sciences, Tianjin University, No. 92 Weijin Road, Nankai District, Tianjin 300072, China

b. Tianjin Key Laboratory of Function and Application of Biological Macromolecular Structures, School of Life Sciences, Tianjin University, Tianjin 300072, China

c. Department of Biological Sciences, University of Toronto Scarborough, 1265 Military Trail, Toronto, Ontario, M1C 1A4, Canada

d. Chen Guanxing Dental Clinic, No. 32 ZhengdaRoad, Jiangbei District, Ningbo, Zhejiang Province

e. Hepatobiliary Pancreatic Center, Xuzhou Central Hospital, Xuzhou 221009, China

f. Hepatopancreatobiliary Center, Beijing Tsinghua Changgung Hospital, Tsinghua University，Beijing 100084, China

g. School of Clinical Medicine, Tsinghua University, Beijing 100084, China

h. Department of Oral Maxillofacial-Head and Neck Oncology, Shanghai Ninth People’s Hospital, Shanghai Jiao Tong University School of Medicine, College of Stomatology, Shanghai Jiao Tong University, No. 639, Zhizaoju Rd, Shanghai, 200011, China

^1^These authors contribute equally to this work.

Corresponding authors:

Jin Chang: jinchang@tju.edu.cn.

Xi Yang: Shixin16@hotmail.com

Jun Yan: yanjun1619@tsinghua.edu.cn

Huansong Li: heiants666@163.com

Jun Kang: jun.kang@tju.edu.cn

**
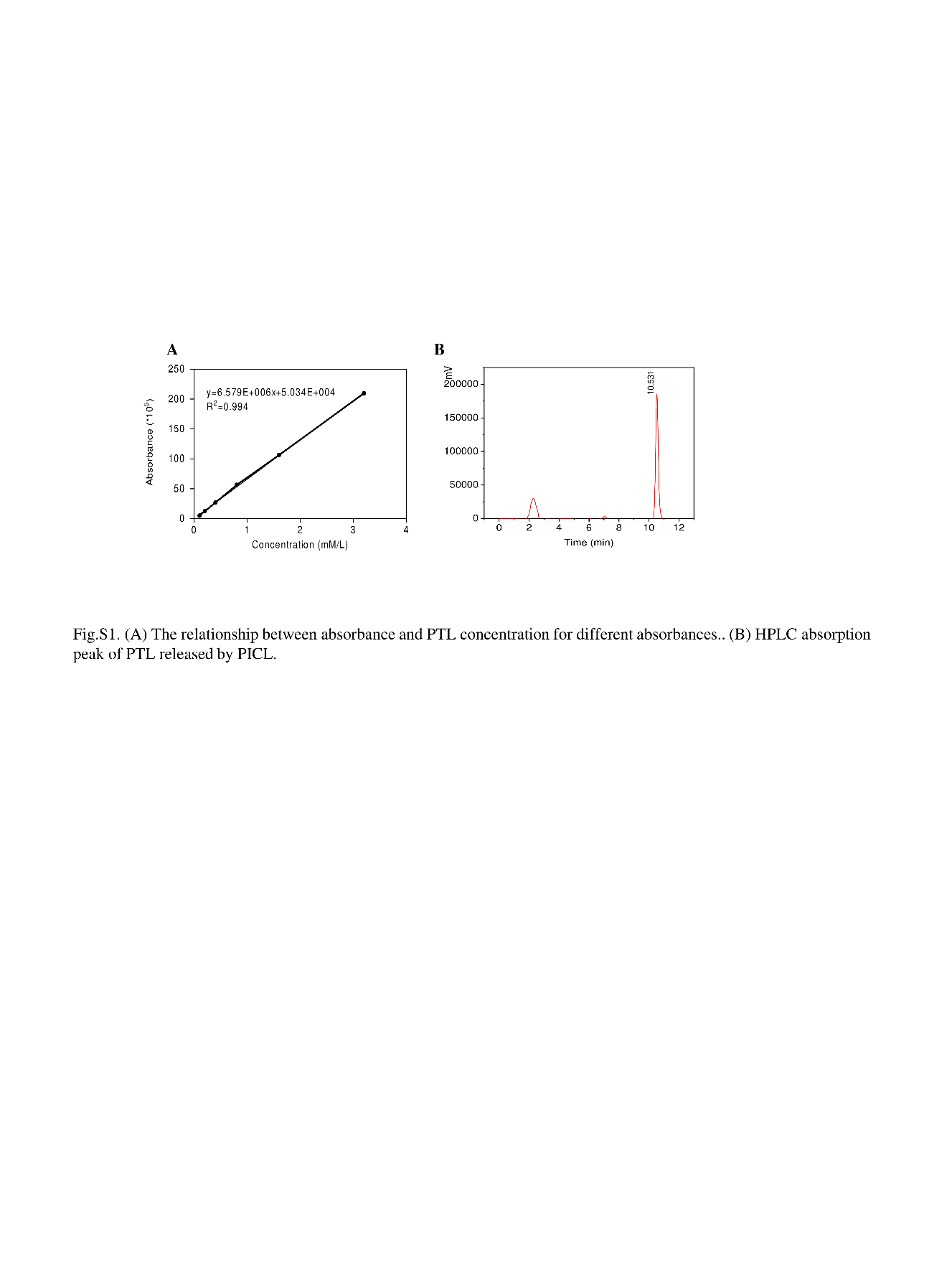
**

**Figure S1.** (A) The relationship between absorbance and PTL concentration for different absorbances. (B) HPLC absorption peak of PTL released by PICL.


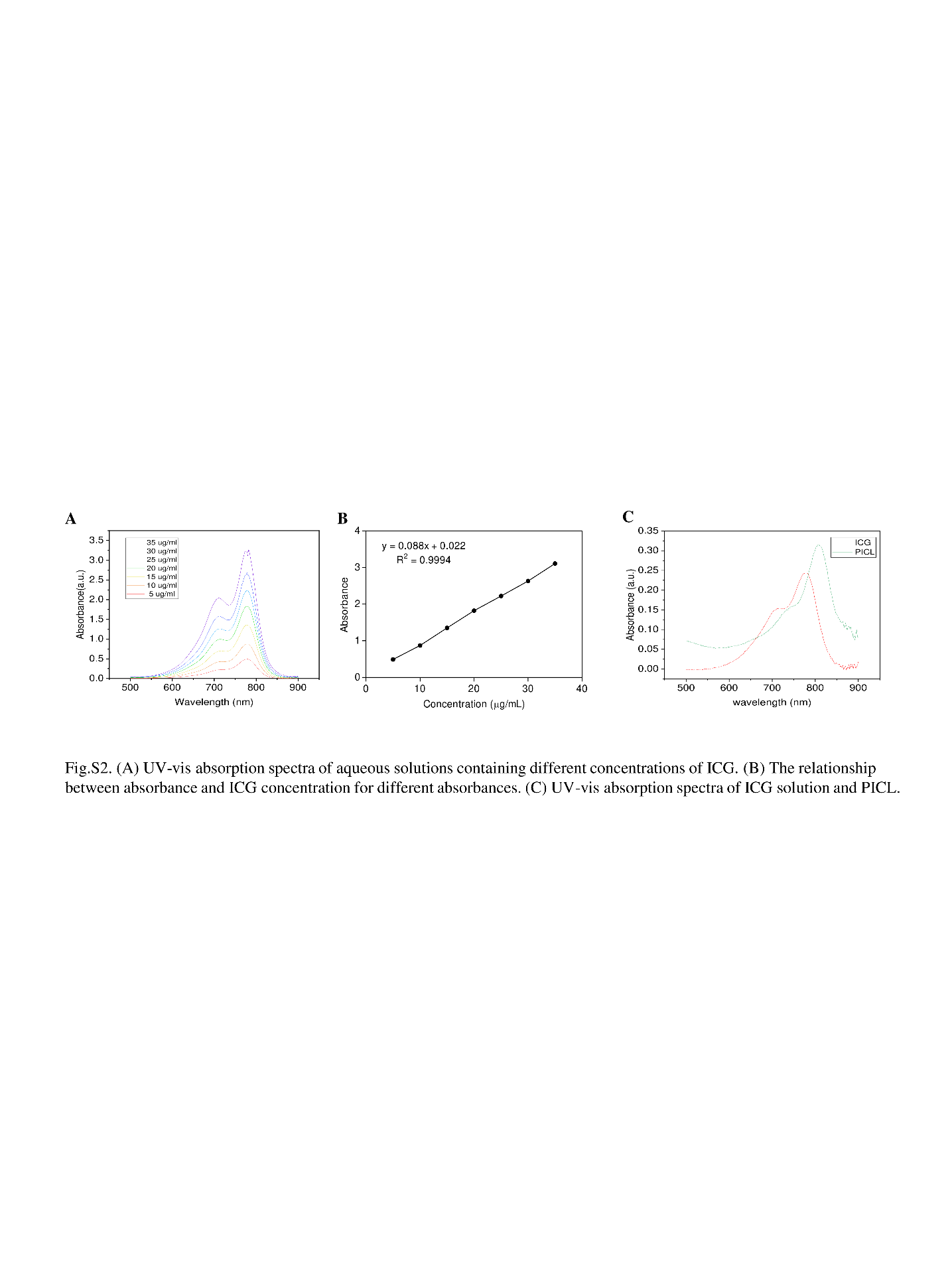


**Figure S2.** (A) UV-vis absorption spectra of aqueous solutions containing different concentrations of ICG. (B) The relationship between absorbance and ICG concentration for different absorbances. (C) UV-vis absorption spectra of ICG solution and PICL.


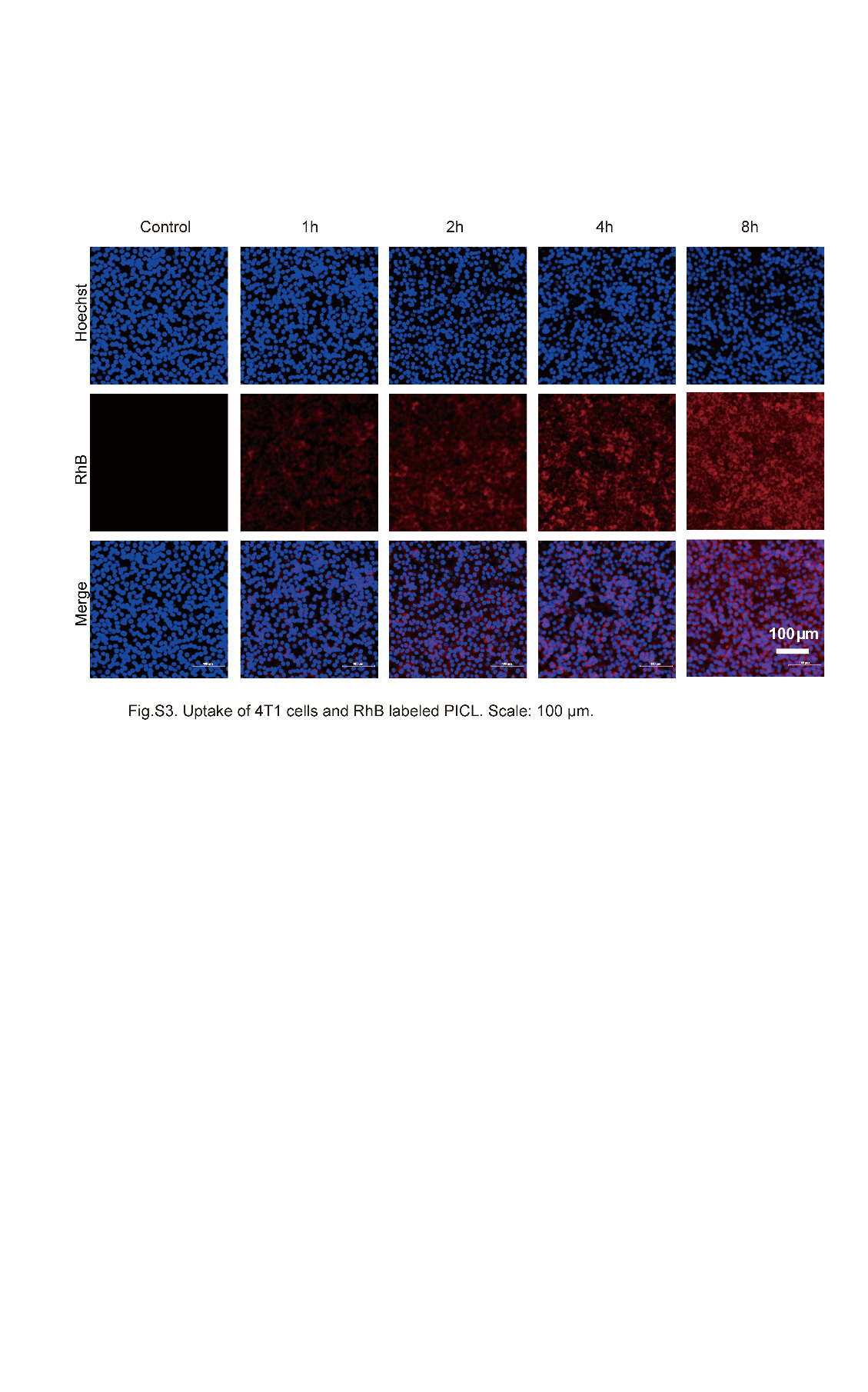


**Figure S3.** Uptake of 4T1 cells and RhB labeled PICL. Scale: 100 μm.


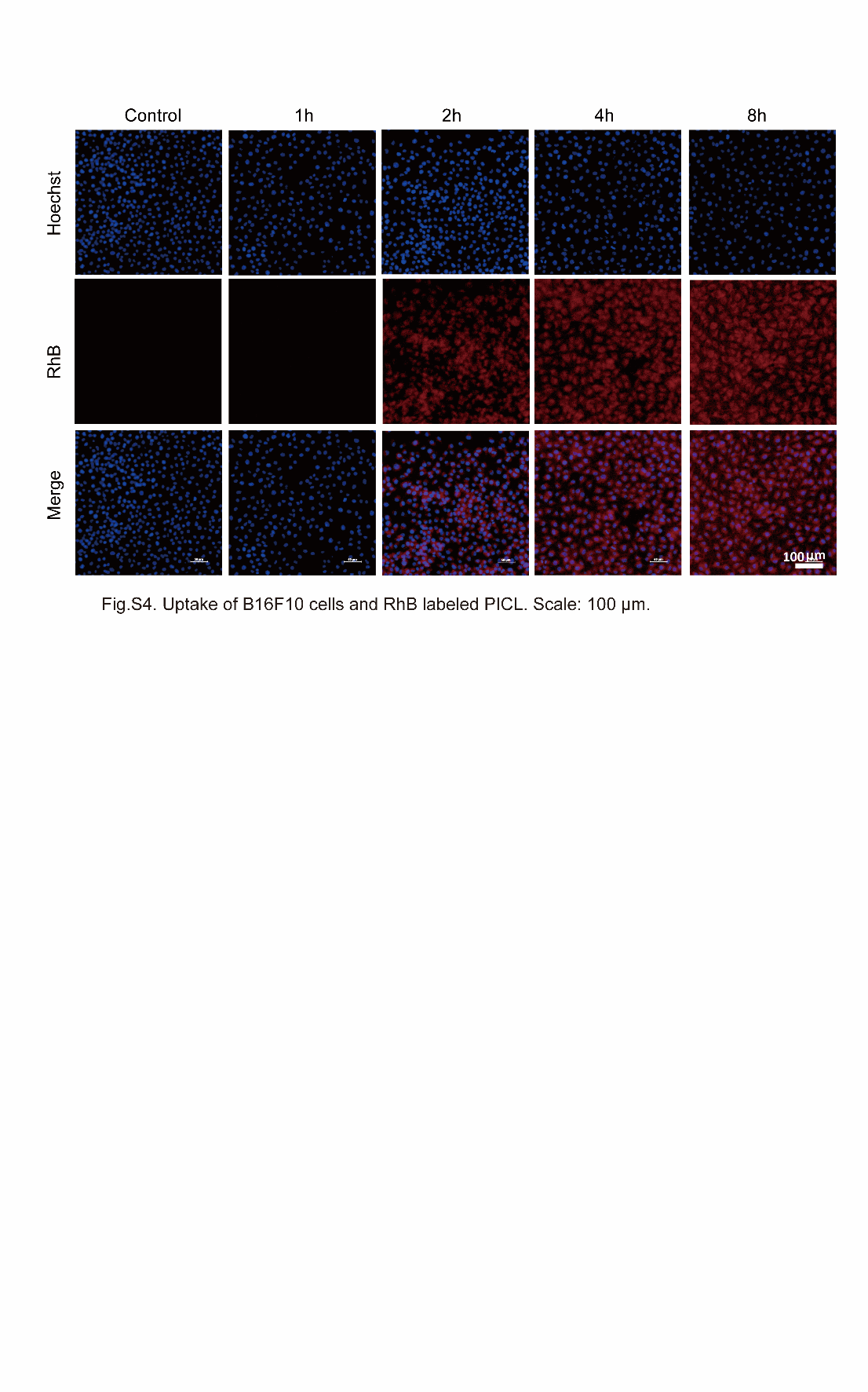


**Figure S4**. Uptake of B16F10 cells and RhB labeled PICL. Scale: 100 μm.

**
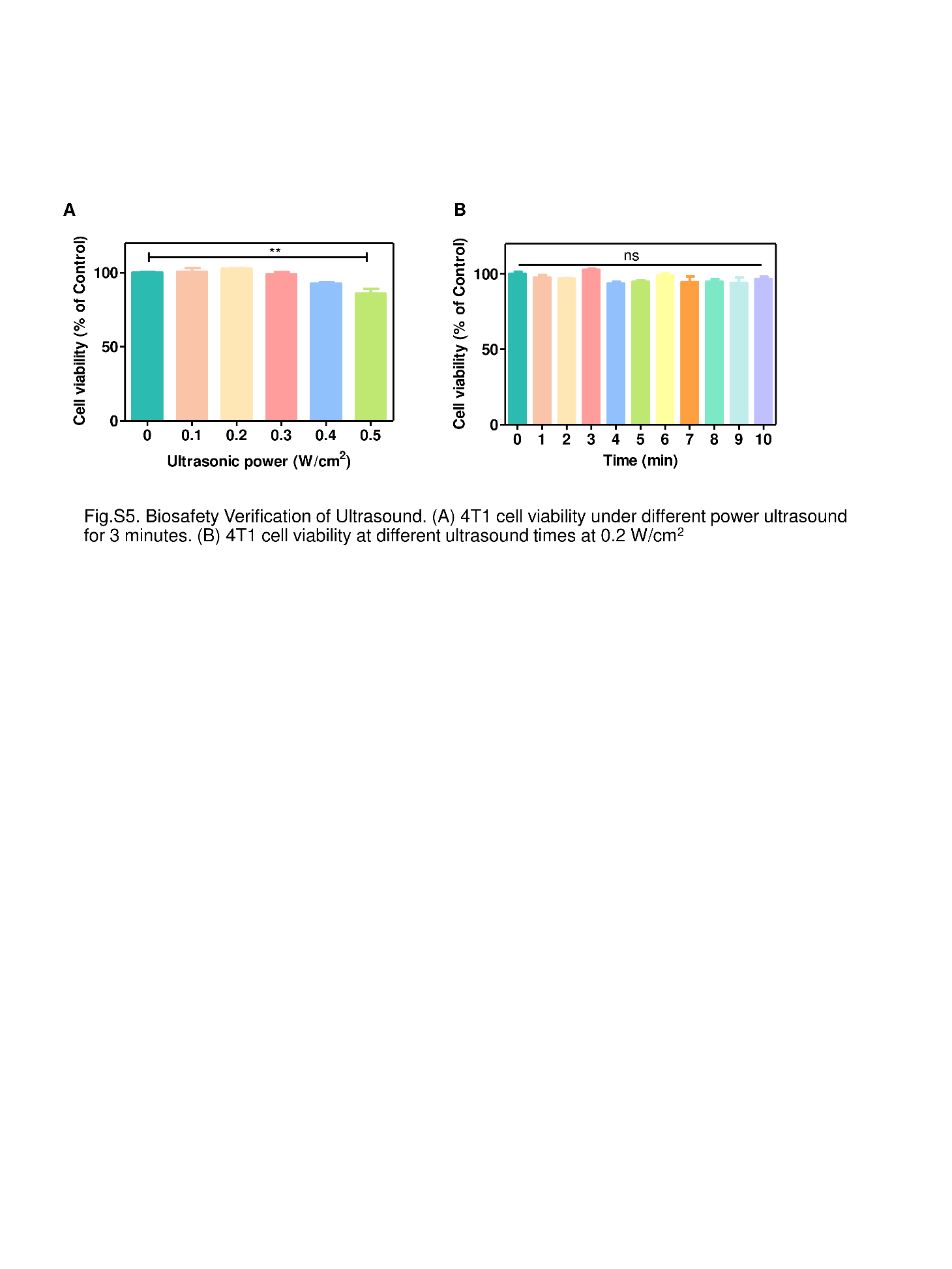
**

**Figure S5**. Biosafety Verification of Ultrasound. (A) 4T1 cell viability under different power ultrasound for 3 minutes. (B) 4T1 cell viability at different ultrasound times at 0.2W cm^-2^


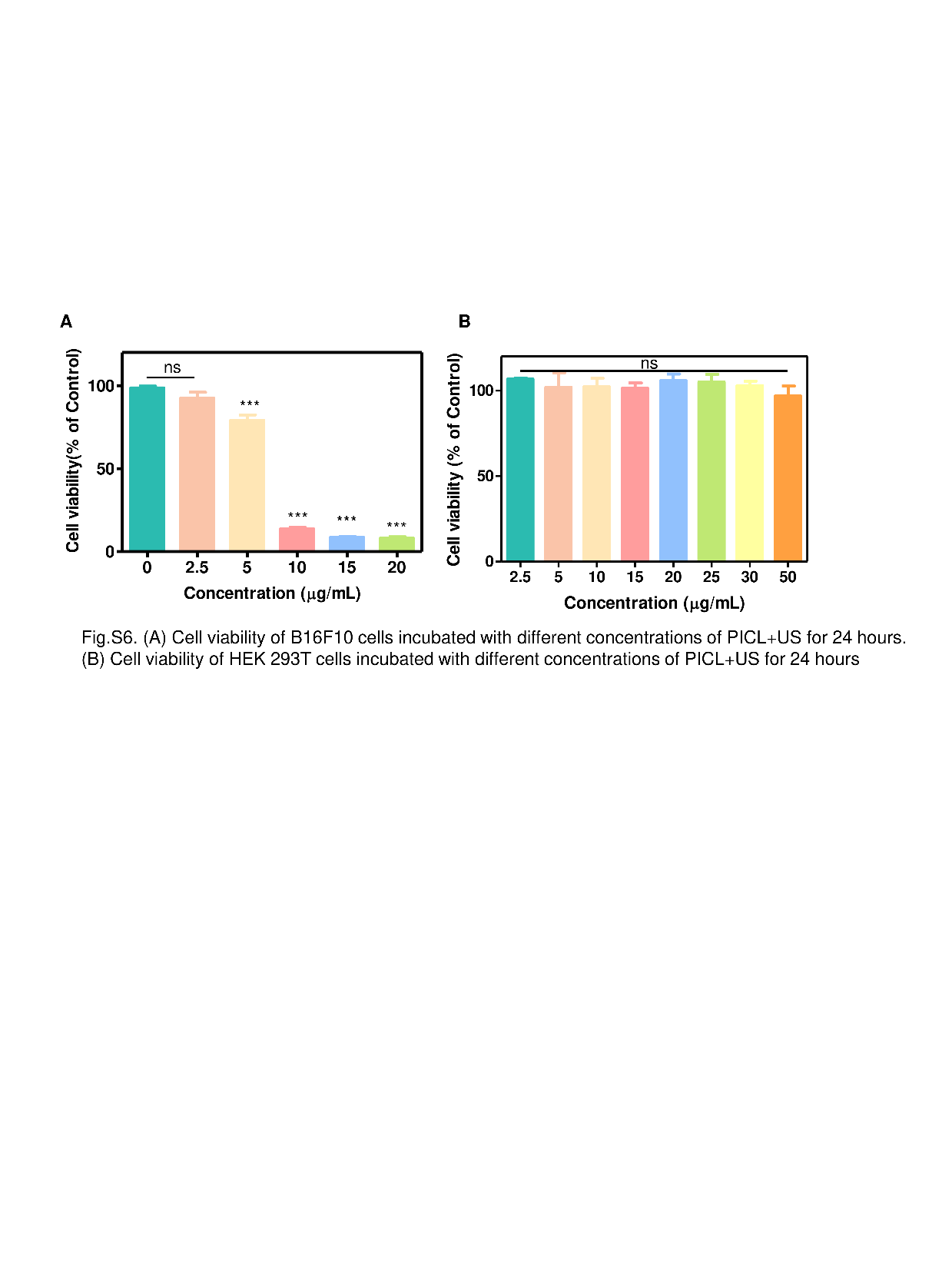


**Figure S6.** Cytotoxicity of PICL. n=3. Cell viability of (A) B16F10 cells and (B) HEK 293T cells incubated with different concentrations of PICL+US for 24 hours.
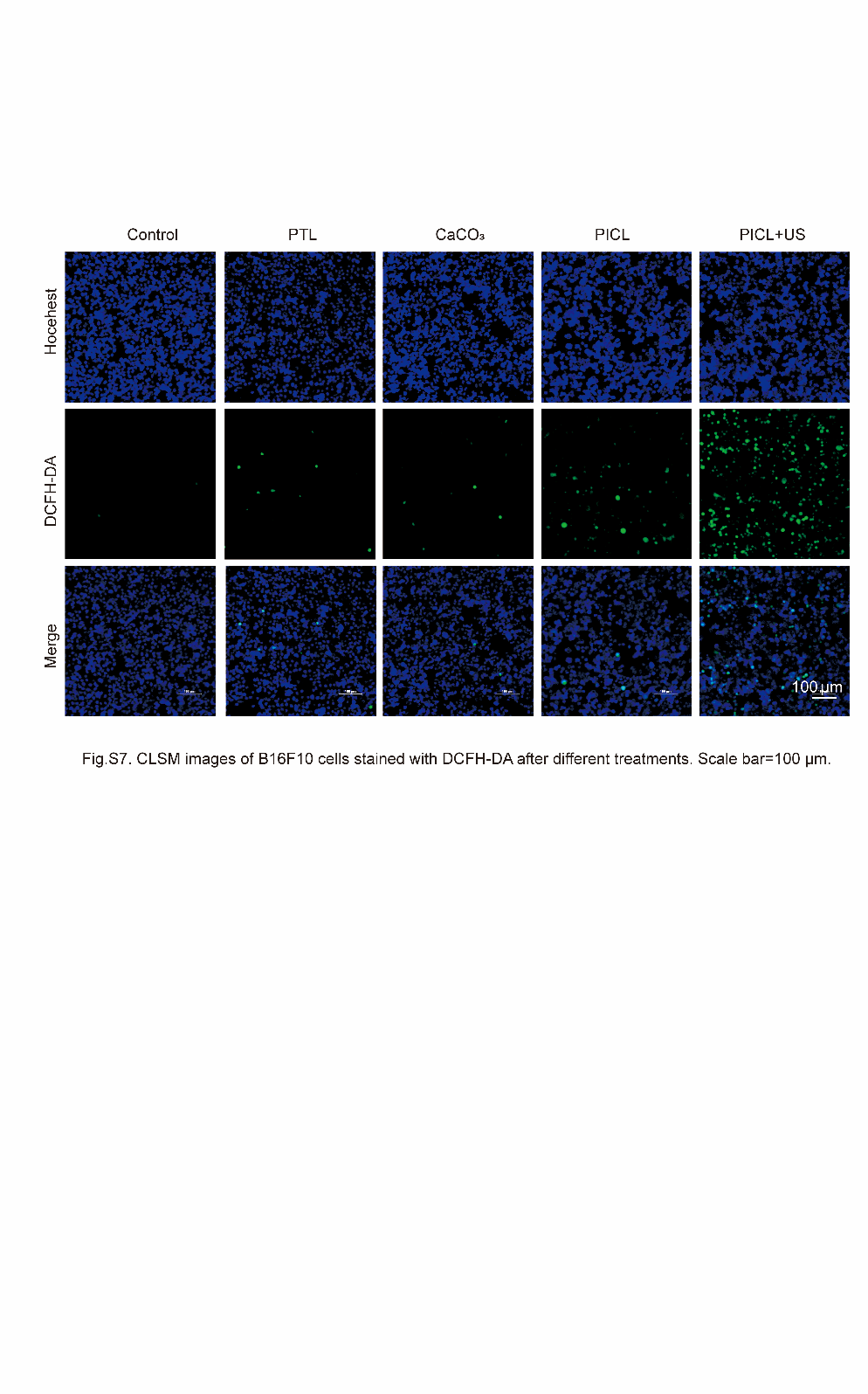


**Figure S7.** CLSM images of B16F10 cells stained with DCFH-DA after different treatments. Scale bar=100 μm.

**
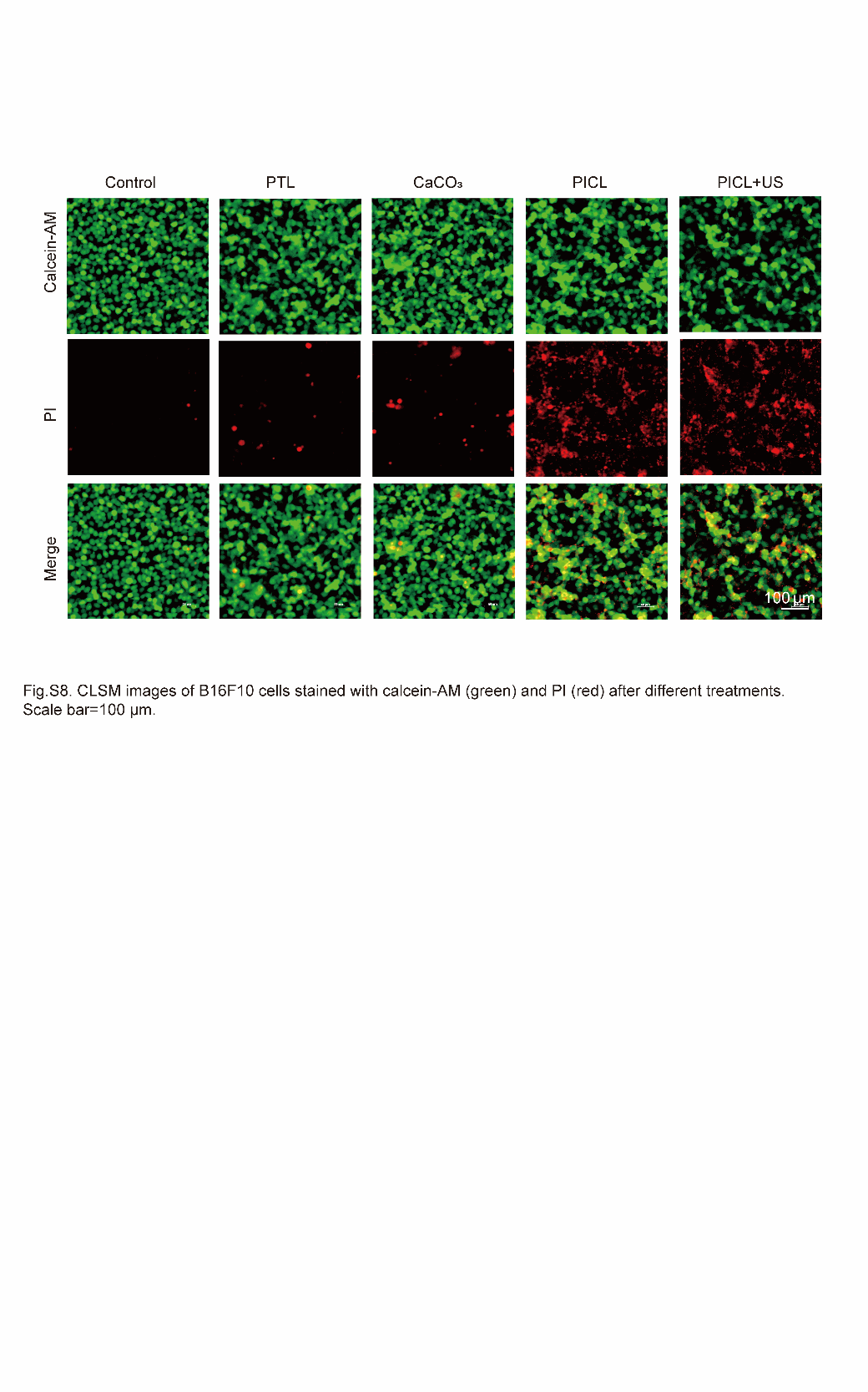
**

**Figure S8.** CLSM images of B16F10 cells stained with calcein-AM (green) and PI (red) after different treatments. Scale bar=100 μm.

Supplemental Table S1. Primer sequence for qRT-PCR

| Gene name | Upstream primer (5’—3’) | Downstream primer (5’—3’) |
| --- | --- | --- |
| β-actin | GGCTGTATTCCCCTCCATCG | CCAGTTGGTAACAATGCCATGT |
| SLC7A11 | GGCACCGTCATCGGATCAG | CTCCACAGGCAGACCAGAAAA |
| LPCAT3 | GACGGGGACATGGGAGAGA | GTAAAACAGAGCCAACGGGTAG |
| Trp53 | GCGTAAACGCTTCGAGATGTT | TTTTTATGGCGGGAAGTAGACTG |


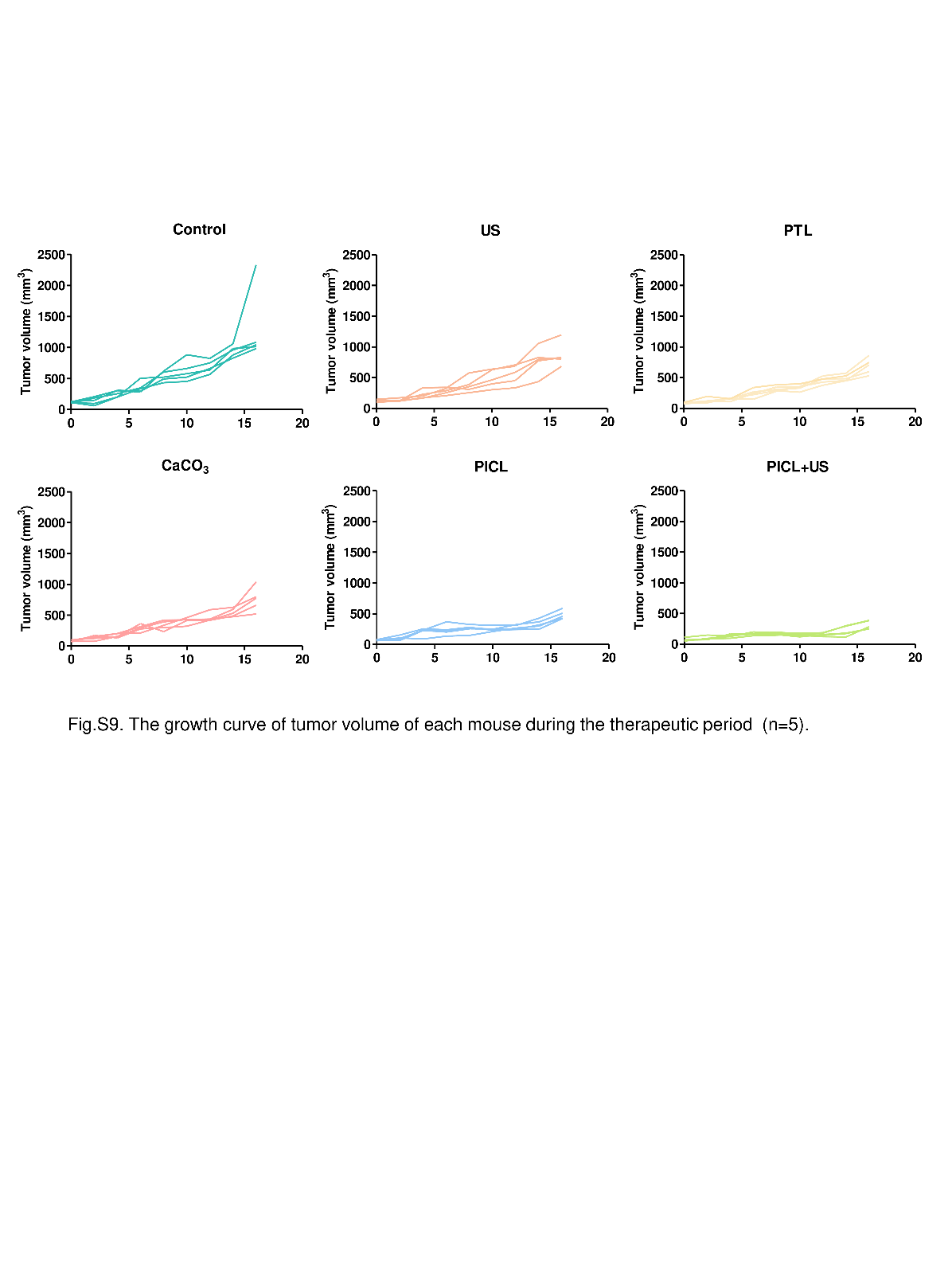


**Figure S9**. The growth curve of tumor volume of each mouse during the therapeutic period.


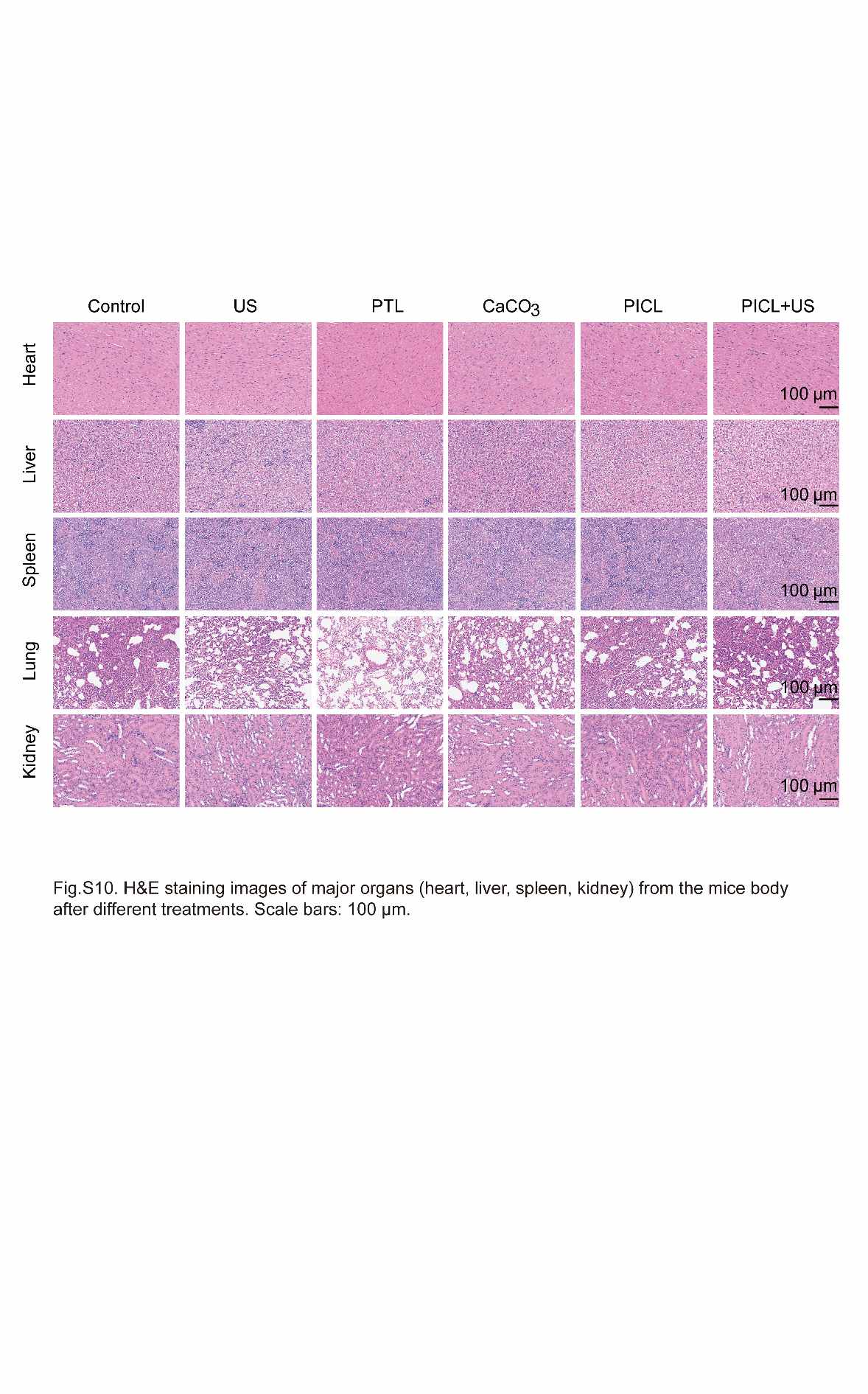


**Figure S10.** H&E staining images of major organs (heart, liver, spleen, kidney) from the mice body after different treatments. Scale bars: 100 μm.


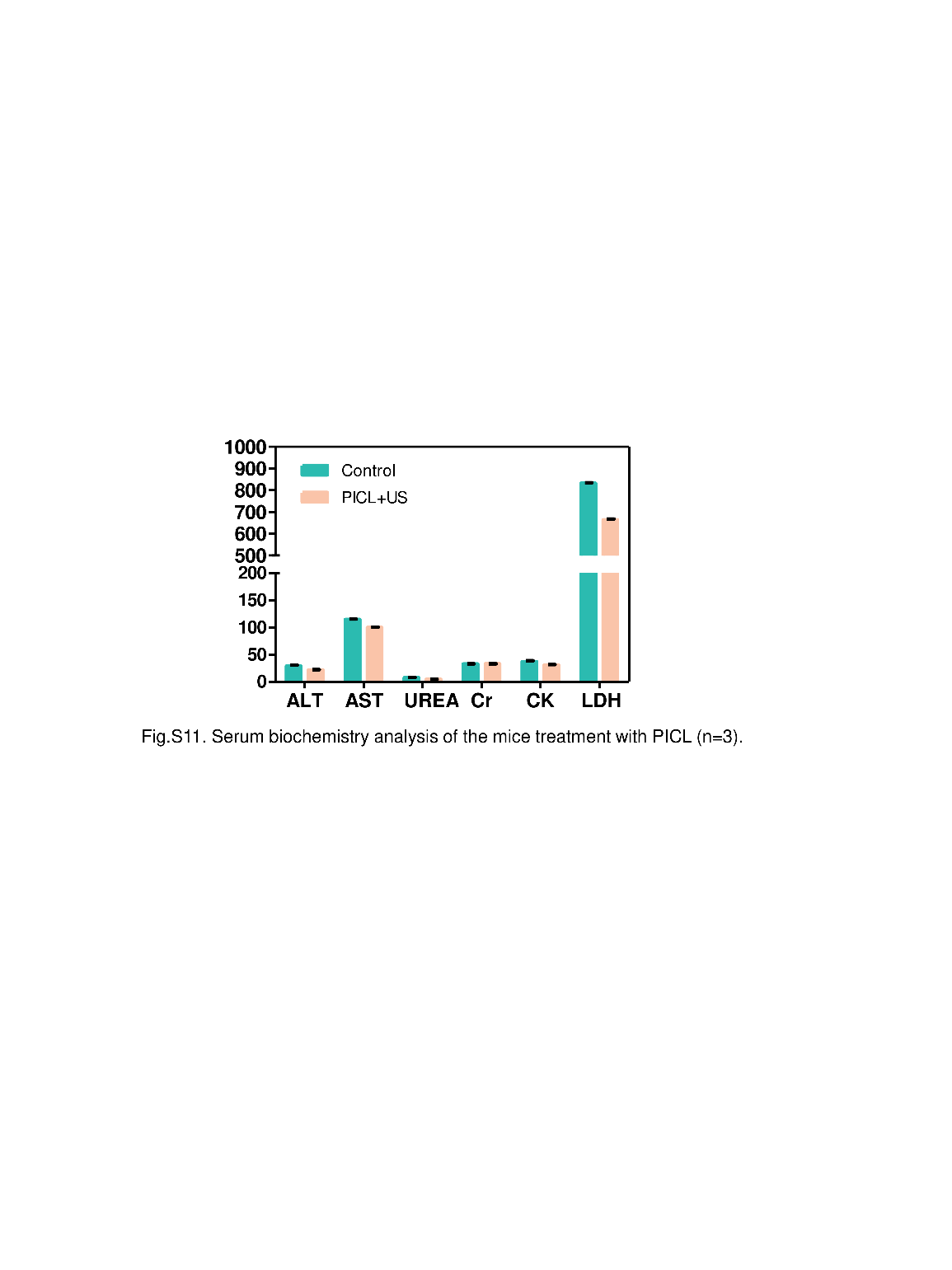
 **Figure S11.** Serum biochemistry analysis of the mice treatment with PICL.
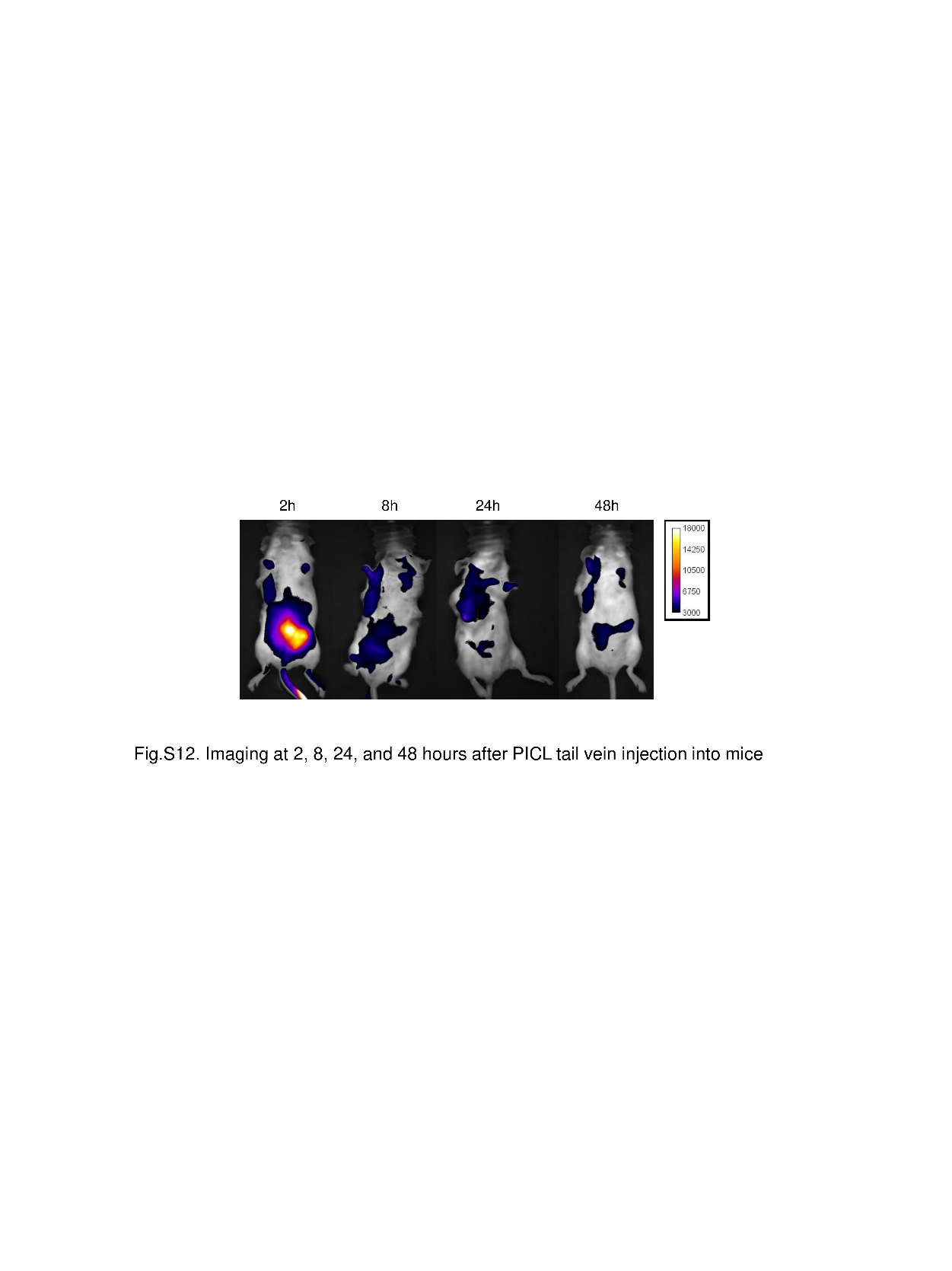


**Figure S12**. Imaging at 2, 8, 24, and 48 hours after PICL tail vein injection into mice.


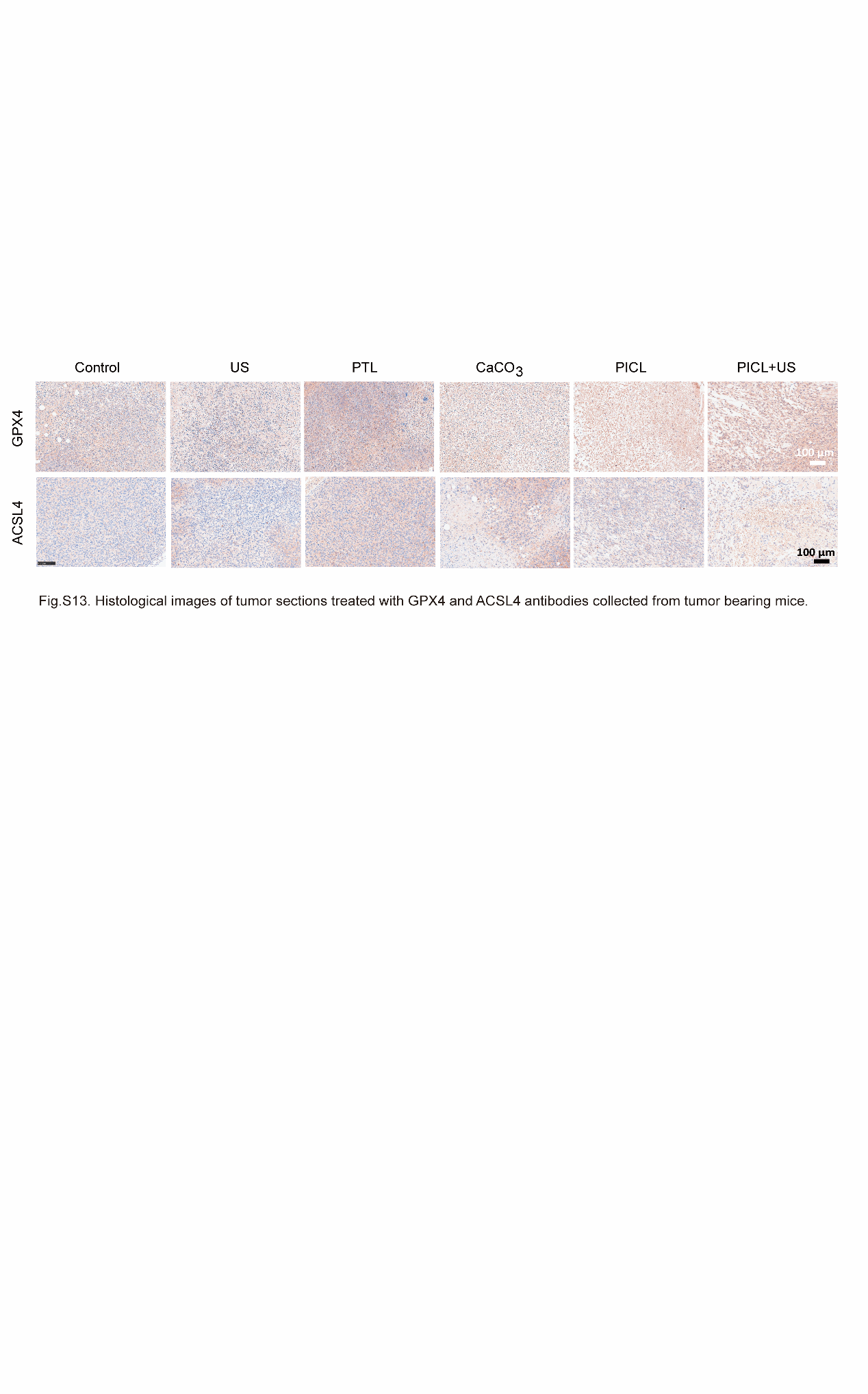


**Figure S13**. Histological images of tumor sections treated with GPX4 and ACSL4 antibodies collected from tumor bearing mice.


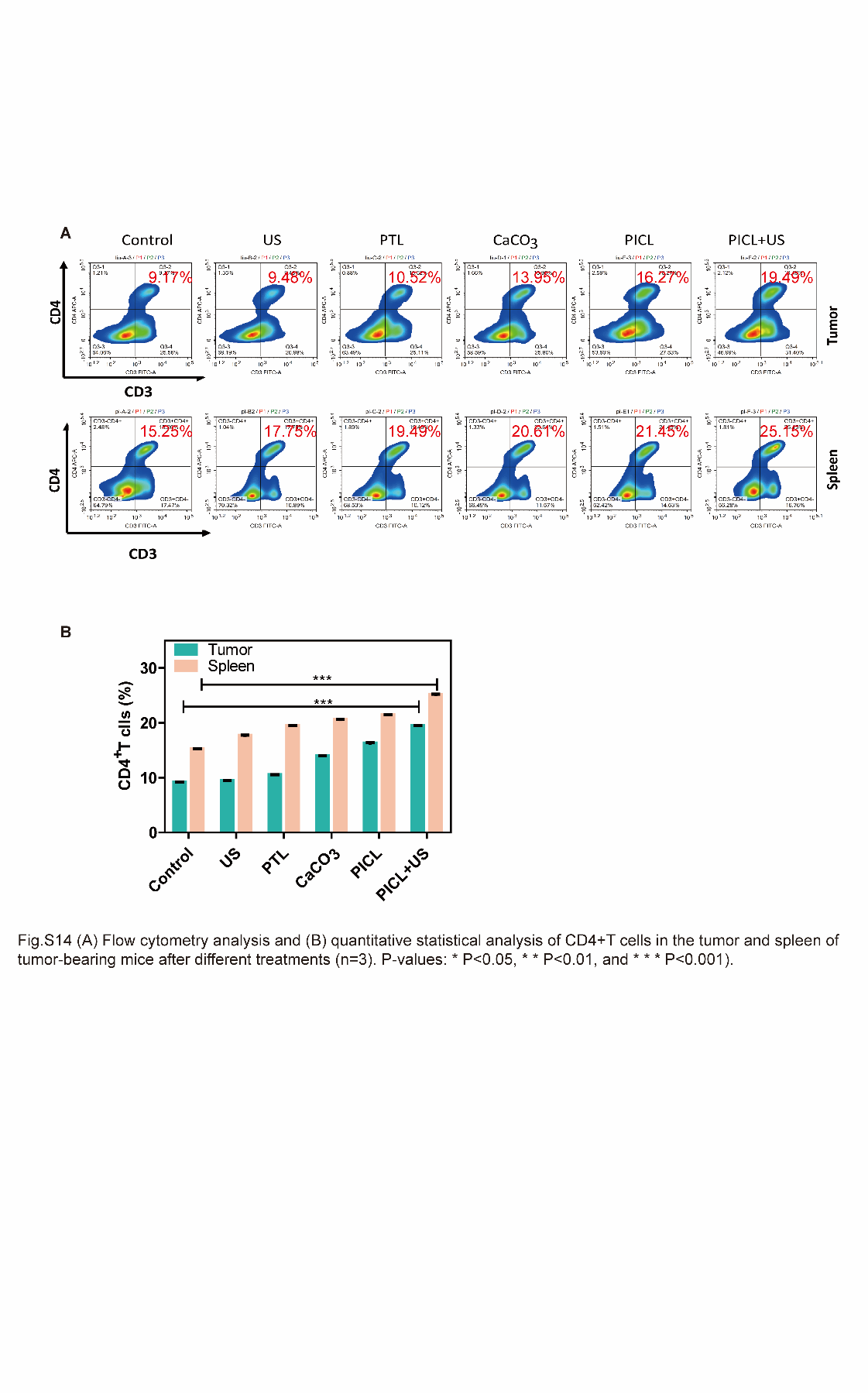


**Figure S14**. (A) Flow cytometry analysis and (B) quantitative statistical analysis of CD4+T cells in the tumor and spleen of tumor-bearing mice after different treatments (n=3). P-values: * P<0.05, * * P<0.01, and * * * P<0.001).
